# Recomendaciones preventivas cardiovasculares. Actualización PAPPS 2020^☆^

**DOI:** 10.1016/j.aprim.2020.08.002

**Published:** 2020-12-30

**Authors:** Domingo Orozco-Beltrán, Carlos Brotons Cuixart, José Juan Alemán Sánchez, José Ramón Banegas Banegas, Ana M. Cebrián-Cuenca, Vicente F. Gil Guillen, Enrique Martín Rioboó, Jorge Navarro Pérez

**Affiliations:** a Unidad de Investigación CS Cabo Huertas, Departamento San Juan de Alicante, Departamento de Medicina Clínica. Universidad Miguel Hernández, España; b Equipo de Atención Primaria Sardenya, Barcelona, España; c Dirección General de Salud Pública, Servicio Canario de la Salud, España; d Facultad de Medicina, Universidad Autónoma de Madrid, Madrid, España; e Centro de Salud Cartagena Casco Antiguo, Cartagena, Murcia, España; f Departamento de Medicina Clínica, Universidad Miguel Hernández, España; g UGC Poniente en Córdoba Distrito Sanitario Córdoba Guadalquivir, Córdoba, España; h Hospital Clínico Universitario, Departamento de Medicina, Universidad de Valencia, Instituto de Investigación INCLIVA, Valencia, España

**Keywords:** Enfermedades cardiovasculares, Diabetes mellitus, Atencion primaria, Medicina de familia, Preventive activities, Cardiovascular diseases, Diabetes mellitus, Primary care, Family medicine

## Abstract

Se presentan las recomendaciones del Programa de Actividades Preventivas y Promoción de la Salud (PAPPS) de la semFYC, para la prevención de las enfermedades cardiovasculares (CV). Se incluyen los siguientes apartados: Revisión epidemiológica, donde se describe la morbimortalidad CV actual en España y su evolución y los principales factores de riesgo; Tablas de riesgo CV y recomendaciones para el cálculo del riesgo CV; Factores de riesgo mayores como hipertensión arterial, dislipidemia y diabetes mellitus, describiendo el método para su diagnóstico, los objetivos terapéuticos y las recomendaciones de medidas de estilo de vida y de tratamiento farmacológico; Indicaciones de antiagregación, y Recomendaciones para el cribado de la fibrilación auricular. Para las principales recomendaciones se incluyen tablas específicas que recogen la calidad de la evidencia y la fuerza de la recomendación.

## Abstract (en)

The recommendations of the semFYC's Program for Preventive Activities and Health Promotion (PAPPS) for the prevention of cardiovascular diseases (CVD) are presented. The following sections are included: Epidemiological review, where the current morbidity and mortality of CVD in Spain and its evolution as well as the main risk factors are described; Cardiovascular (CV) risk tables and recommendations for the calculation of CV risk; Main risk factors such as arterial hypertension, dyslipidemia and diabetes mellitus, describing the method for their diagnosis, therapeutic objectives and recommendations for lifestyle measures and pharmacological treatment; Indications for antiplatelet therapy, and recommendations for screening of atrial fibrillation. The quality of testing and the strength of the recommendation are included in the main recommendations.

## Revisión epidemiológica

### Mortalidad cardiovascular

En 2018, 120.859 personas murieron en España por enfermedades cardiovasculares (CV), que siguen siendo la primera causa de muerte (28,3%), seguido por los tumores (26,4%) y las enfermedades respiratorias (12,6%). Sin embargo, dentro del conjunto de causas, la mortalidad CV se ha reducido del 34,9% en el año 2000 (30,1% en hombres y 40,2% en mujeres) al 28,3% en 2018 (25,9% en hombres y 30,7% en mujeres)^1^.

En el año 2018, la enfermedad isquémica representaba el 7,3% y las enfermedades cerebrovasculares el 6,2% del total de la mortalidad^2^. La preponderancia de la enfermedad isquémica del corazón sobre la cerebrovascular se produce a expensas de los varones (9,1 vs. 5,5 en 2016 y 8,5 vs. 5,3 en 2018), dato que ya no ocurre en las mujeres (6,4% vs. 7,7% en 2016 y 6,0% vs. 7,1% en 2018).

Los factores de riesgo más importantes, por su prevalencia e impacto en la salud son el tabaco, la presión arterial (PA) elevada, el índice de masa corporal (IMC) elevado, el consumo de alcohol y la glucemia elevada^2^.

El riesgo de morir (tasas ajustadas por edad) por las enfermedades CV está disminuyendo en España desde mediados de la década de 1970, sobre todo debido al descenso de la mortalidad cerebrovascular. Sin embargo, a causa del envejecimiento de la población, a pesar de que las tasas ajustadas de mortalidad por coronariopatía han disminuido en este período, el número de muertes ha incrementado, por lo que el impacto sanitario y social de estas enfermedades está aumentando. La tasa estandarizada de mortalidad CV fue en 2018 de 230,5/10^5^ habitantes, pero con una importante diferencia entre comunidades autónomas (Madrid 168,8/10^5^ vs. Andalucía 300,4/10^5^).

En el ámbito internacional, las tasas de mortalidad ajustadas por edad de España para el conjunto de las enfermedades del sistema circulatorio, la enfermedad isquémica del corazón y para la enfermedad cerebrovascular son más bajas que en otros países occidentales^2^.

### Morbilidad cardiovascular

En el año 2018, en España, la tasa de morbilidad hospitalaria de las enfermedades del sistema circulatorio fue de 1.310 por 10^5^ habitantes (1.521 en varones y 1.107 en mujeres)^1^ y causó 5,4 millones de estancias hospitalarias y 612.066 altas. La tasa de morbilidad hospitalaria de la enfermedad isquémica del corazón fue de 223 por 10^5^ habitantes (335 en los varones y 115 en las mujeres). Respecto a la enfermedad cerebrovascular, la tasa de morbilidad fue de 236 por 10^5^ habitantes (260 en varones y 213 en mujeres). Por tanto, se observa que la morbilidad por enfermedad isquémica del corazón es superior a la cerebrovascular en los varones, mientras que en las mujeres ocurre lo contrario. La tendencia de las tasas de morbilidad hospitalaria de las enfermedades del sistema circulatorio ha sido de un constante aumento desde 1977 hasta 2003, con un ligero descenso en el período 2003-2012 y, a partir de ese año, sigue aumentando.

### Años potenciales de vida perdidos

Un aspecto que merece destacarse es que las enfermedades CV siguen siendo la primera causa de mortalidad prematura (< 70 años) en toda Europa y también en España^3^. Del total de años potenciales de vida perdidos, las enfermedades CV supusieron en Europa el 34% en hombres y 37% en mujeres, mayor que el cáncer, que supuso un 22% en hombres y un 25% en mujeres^3^.

### Factores de riesgo cardiovascular

La hipertensión arterial (HTA), la dislipidemia y la diabetes son factores de riesgo mayores y causales de enfermedad CV y mortalidad vascular y total^2^. La carga de estos factores de riesgo CV en la población española se describe en el Estudio de Nutrición y Riesgo Cardiovascular en España (ENRICA), que se realizó en el año 2010^4^. Se trata de un estudio descriptivo, transversal, de base poblacional, representativo de la población general española mayor de 18 años, en el que se establecen la prevalencia, el conocimiento, el tratamiento y el control de cada uno de los principales factores de riesgo CV.

### Hipertensión arterial

La HTA, definida como unas cifras de PA sistólica (PAS)/diastólica (PAD) obtenidas de forma protocolizada en la clínica > 140/90 mmHg, o por estar tratado con medicamentos antihipertensivos, es un problema de salud pública importante por su alta prevalencia en muchos países, especialmente en las personas mayores. Además, la HTA está todavía insuficientemente controlada en la mayoría de poblaciones y ámbitos clínicos^5^, ^6^. Según el estudio di@bet.es, la prevalencia de la HTA en la población general adulta de España es del 43% (49,9% en hombre y 37,1% en mujeres)^5^. Aproximadamente, el 40% de las personas con hipertensión desconoce que la presentan (43% en hombres y 32% en mujeres), solo el 12% de los diagnosticados no está tratado con medicamentos antihipertensivos pero el 70% de los tratados no alcanza objetivos terapéuticos de control (< 140/90 en población general o < 130/80 en pacientes con diabetes, nefropatía o enfermedad CV)^5^, ^6^. Por ello, solo una de cada 7 personas con hipertensión en la población general está adecuadamente controlada^5^. En el estudio PRESCAP en personas mayores de 60 años, el uso de monitorización ambulatoria de la PA (MAPA) permitió identificar a más pacientes con buen control (37.4% vs. 54.1%) que con la medición de la PA ambulatoria tradicional^7^.

Del mismo modo, basada en MAPA, la prevalencia de hipertensión es apreciablemente inferior y el control mayor debido a la ausencia de la reacción de alerta o bata blanca^8^. Según datos del estudio DARIOS, realizado en 10 comunidades autónomas de España, se evidencia poca variación geográfica en la frecuencia de hipertensión en personas de 35 a 74 años^9^. Además, la prevalencia de enfermedad CV y de lesiones de órganos diana en las personas con hipertensión es superior a la de la población general y afecta a 1 de cada 4 pacientes^10^.

### Dislipidemia

La hipercolesterolemia, que se considera con las cifras de colesterol total > 200 mg/dl, o bien por tener un tratamiento farmacológico hipolipidemiante, es el factor de riesgo más prevalente y afecta al 50% de la población adulta española^4^, ^11^. Sin embargo, es el menos conocido (50%), el menos tratado (42%) y, aunque ha mejorado, el control sigue siendo moderado (< 50%)^3^, ^12^. Un 26% de la población tiene un colesterol unido a lipoproteínas de alta densidad (cHDL) < 50 mg/dl^9^. La elevada prevalencia de la hipercolesterolemia persiste aun para cifras de colesterol total > 250 mg/dl: el 43% de los varones y el 40% de las mujeres en población de entre 35 y 74 años^11^.

### Diabetes mellitus tipo 2

La prevalencia de diabetes en España en población general > 18 años es de un 7-13%, según algunos estudios de base poblacional en España^4^, ^13^, mayoritariamente conocida (79%), tratada con fármacos (85%) y controlada (69%)^4^. Un estudio poblacional en Cataluña^14^ muestra que solo un 13% de los pacientes diabéticos tipo 2 que estaban en prevención primaria tenía buen control de los principales factores de riesgo CV (hemoglobina glucosilada [HbA1c] < 7%, PA < 130/80 mmHg y colesterol unido a lipoproteínas de baja densidad [cLDL] < 130 mg/dl), y que un 12% de los que estaban en prevención secundaria tenía buen control (HbA1c < 7%, PA < 130/80 mmHg y cLDL < 100 mg/dl).

### Efecto agregado de factores de riesgo

La prevalencia de síndrome metabólico en adultos en España, según el estudio DARIOS, es del 31%, algo mayor en varones que en mujeres (el 32 y el 29%, respectivamente) y está asociado a mayor riesgo coronario^15^. Finalmente, basado en los principales factores de riesgo CV, el riesgo CV en España es moderado en el 56% de los varones y bajo en el 55% de las mujeres^16^. Sin embargo, el grado de control de estos factores en sujetos de alto riesgo es escaso.

## Tablas de riesgo cardiovascular

### Importancia epidemiológica y clínica

Las tablas o funciones de riesgo nos permiten estimar el riesgo absoluto de presentar un evento CV en un período de 10 años. Es decir, si el riesgo CV de una persona es del 5%, de 100 personas con su perfil de riesgo, 5 presentarían un evento CV en los próximos 10 años. Las Guías Europeas de Prevención Cardiovascular y el Comité Español para la Prevención Vascular (CEIPV) recomiendan la utilización de las tablas SCORE para países de bajo o alto riesgo, pero también recomiendan el uso de tablas de ámbito nacional, si han sido adecuadamente calibradas y validadas^17^, ^18^, ^19^.

Se ha publicado que en población española las tablas SCORE para países de bajo riesgo sobrestiman considerablemente el riesgo^20^, ^21^ y su capacidad predictiva en pacientes con hipercolesterolemia es limitada^22^.

Existen experiencias de recalibración de la ecuación de Framingham de 1998 con el REGICOR^23^ y de su validación en las cohortes del estudio VERIFICA (Validez de la Ecuación de Riesgo Individual de Framingham de Incidentes Coronarios Adaptada)^24^. También se han desarrollado a partir de 11 cohortes españolas las tablas FRESCO^25^, que son precisas y fiables para la predicción del riesgo de enfermedad coronaria e ictus a los 10 años, en población de 35 a 79 años. Es muy importante ir recalibrando las tablas que se utilicen, adaptándolas a los cambios en la prevalencia de los factores de riesgo y la incidencia de enfermedad CV. Por ejemplo, la utilización del REGICOR sobrestima ligeramente el riesgo en la población FRESCO.

Hay otros aspectos importantes en la evaluación del riesgo. Primero, la necesidad de desarrollar tablas de riesgo para pacientes que ya hayan presentado una enfermedad CV, ante la aparición de nuevos y costosos tratamientos, como los inhibidores de la proproteína convertasa subtilisina/kexina tipo 9 (PCSK9), y porque los predictores de riesgo pueden ser muy diferentes de los de prevención primaria. Segundo, la utilización del riesgo CV de por vida en pacientes jóvenes que difícilmente llegan a ser de alto riesgo, para cuyo cálculo, desde los 18 hasta los 75 años, se ha desarrollado un modelo, a partir de población laboral española (IBERLIEFRISK), que permite calcular el riesgo desde los 18 hasta los 75 años^26^, y actualmente se está trabajando en su validación externa. Tercero, el reto de la comunicación del riesgo y la toma de decisiones compartidas en la práctica clínica. Además de la edad vascular y el riesgo relativo (RR), se han publicado nuevos abordajes para calcular el beneficio a largo plazo y los años de vida ganados con fármacos para el control de la dislipidemia y la HTA, antiagregantes y abandono del consumo de tabaco^27^, ^28^. Y cuarto, la utilización de los datos basales de los estudios de cohortes que se han utilizado para desarrollar los diferentes modelos son muy simplistas, ya que la realidad es que todos los posibles predictores, como la PA, el colesterol, el peso, los tratamientos farmacológicos y otros, no son estáticos y van cambiando a lo largo del tiempo de seguimiento. Una línea de investigación abierta y con mucho futuro es lo que se denomina Machine Learning^29^, que permite analizar la relación entre predictores y eventos de forma más ajustada con modelos más complejos que los basados en una simple relación lineal entre el valor basal y el evento 10 años después.

#### Factores de riesgo no convencionales

Las tablas incluyen un número reducido de factores de riesgo, pero se han descrito otros que podrían ser útiles para modificar el riesgo calculado con las tablas.

Para considerar un factor de riesgo útil se requiere que: *a)* sea capaz de reclasificar adecuadamente el riesgo; *b)* no exista sesgo de publicación; *c)* su medición sea coste-efectiva. Las guías europeas incluyen el nivel socioeconómico, la historia familiar de enfermedad CV prematura, la obesidad (central), el índice tobillo brazo, la presencia de placas en las arterias carótidas y la puntuación de calcio coronario^17^. Las guías norteamericanas incluyen estos y otros modificadores del riesgo^30^, pero la evidencia sobre su utilidad en la práctica clínica es limitada. El biomarcador con mayor capacidad predictiva es el calcio intracoronario, pero se considera una exploración adicional innecesaria por la relación coste-beneficio y el riesgo de radiación^31^.

Las guías europeas de prevención CV establecen una serie de categorías para la estratificación del riesgo CV. En la tabla 1 se muestran estas categorías adaptadas de las recomendaciones Comité Español Interdisciplinario para la Prevención Vascular de 2020 (CEIPV 2020, en vías de publicación). Como se puede observar, a los pacientes diabéticos ya los considera de riesgo muy alto o alto, por lo que no sería necesario calcular el riesgo CV.

**Tabla 1:** Categorías de riesgo

Riesgo muy alto	Enfermedad cardiovascular documentada, ya sea clínicamente o a través de imágenes, incluyendo infarto de miocardio, síndrome coronario agudo, revascularización coronaria o de otras arterias, ictus y accidente vascular transitorio, y enfermedad vascular periférica, así como la presencia de placas en la arteriografía coronaria o en la ecografía carotídea. No incluiría aumento del grosor de la íntima media carotídea Diabetes con afectación de órgano diana como proteinuria o con la presencia de un factor de riesgo mayor, como tabaquismo, hipercolesterolemia o hipertensión ERC grave (FG < 30 ml/min/1,73 m^2^) Puntuación SCORE >10% o REGICOR >15%
Riesgo alto	Un factor de riesgo elevado, como colesterol > 8 mmol/l (310 mg/dl), PA > 180/110 mmHg El resto de personas con diabetes (con la excepción de jóvenes con diabetes mellitus tipo 1 y sin factores de riesgo, que podrían ser de riesgo moderado o bajo) ERC moderada (FG, 30-59 ml/min/1,73 m^2^) Puntuación SCORE 5-10% o REGICOR 10-15%
Riesgo moderado	Puntuación SCORE 1-4% o REGICOR 5-10%
Riesgo bajo	Puntuación SCORE < 1% o REGICOR < 5%

ERC: enfermedad renal crónica; FG: filtrado glomerular; PA: presión arterial.

#### Cálculo del riesgo cardiovascular

En nuestro medio se recomiendan las tablas SCORE para países de bajo riesgo y los factores que se incluyen en las tablas son la edad, el sexo, la PAS, el colesterol total y el tabaco. Se consideraría de riesgo muy alto cuando este resulta ser > 10% de mortalidad CV a los 10 años. Entre el 5 y el 9% se considera alto, entre el 1 y el 4%, moderado, y < 1%, bajo. Actualmente, se puede calcular directamente a través de la web y brinda la posibilidad de incluir el cHDL (https://heartscore.escardio.org), que ofrece una estimación mucho más precisa del riesgo. Otras tablas que se recomiendan en nuestro medio son las tablas REGICOR que incluyen la edad, el sexo, la PAS, la PAD y el colesterol total, y hay tablas específicas para personas con diabetes. Algunos autores sugieren que si el cHDL es < 35 mg/dl, el riesgo debe multiplicarse por 1,5, y si el cHDL es > 60 mg/dl, el riesgo debe multiplicarse por 0,5. También se puede acceder directamente a la web y se puede calcular el riesgo de una forma más precisa (https://www.imim.cat/ofertadeserveis/software-public/regicor). REGICOR estima el riesgo de morbimortalidad coronaria a los 10 años y cuando es > 15% se considera muy alto, entre el 10 y el 14%, alto, entre el 5 y el 9%, moderado y < 5%, bajo.

En la tabla 2 se recoge la calidad de la evidencia y la fuerza de la recomendación sobre el cálculo del riesgo CV.

**Tabla 2:** Recomendaciones para el cálculo del riesgo cardiovascular

	Calidad de la evidencia	Fuerza de la recomendación
Se recomienda el cálculo del riesgo cardiovascular a todos los adultos de 40 años o mayores que no tengan una enfermedad cardiovascular o que, por sus características, no sean de alto riesgo, mediante algún sistema validado, como el SCORE (hasta los 65 años) o REGICOR (hasta los 75 años)	Moderada	Fuerte a favor
Las tablas de riesgo constituyen una información complementaria y útil para ayudar a estratificar el riesgo y a tomar decisiones en el tratamiento de la dislipidemia y de la HTA	Moderada	Fuerte a favor

## Hipertensión arterial

En este apartado, se actualizan las recomendaciones del PAPPS sobre HTA del 2018^32^, siguiendo fundamentalmente las recomendaciones de las últimas guías europeas^33^ y considerando las principales controversias y novedades surgidas desde entonces. Las principales recomendaciones incluyendo la calidad de la evidencia y la fuerza de la recomendación se presentan resumidas en la tabla 3.

**Tabla 3:** Recomendaciones en hipertensión arterial

	Calidad de la evidencia	Fuerza de la recomendación
*Prueba diagnóstica*		
La prueba inicial de cribado es la toma de PA en consulta	Fuerte	Fuerte a favor
Periodicidad del cribado cada 3-5 años	Débil	Fuerte a favor
Periodicidad anual en mayores de 40 años o si hay factores de riesgo para el desarrollo de la HTA (sobrepeso-obesidad, cifras de PA normal-alta, o raza negra)	Débil	Fuerte a favor
Debe confirmarse el diagnóstico de HTA mediante MAPA	Fuerte	Débil a favor
Debe confirmarse el diagnóstico de HTA mediante AMPA	Moderada	Fuerte a favor
*Estilos de vida*		
La pérdida de peso, el ejercicio físico, aeróbico, la reducción del consumo de alcohol y de la sal de la dieta o y la dieta mediterránea reducen la PA	Fuerte	Fuerte favor
*Tratamiento farmacológico*		
Los diuréticos, antagonistas del calcio, IECA, ARA-II y los betabloqueantes son los fármacos de elección para iniciar el tratamiento farmacológico	Fuerte	Fuerte favor
La asociación de los grupos anteriores, sobre todo en un sol comprimido, disminuye la PA de forma más acentuada y la morbimortalidad cardiovascular	Fuerte	Fuerte favor
La asociación IECA-ARA-II (o con inhibidores de renina) y la de bloqueadores beta con antagonistas del calcio no dihidropiridínicos está contraindicada	Fuerte	Fuerte en contra
Los IECA y los ARA-II son los fármacos de elección en el diabético con o sin nefropatía	Fuerte	Fuerte a favor
Iniciar inmediatamente el tratamiento farmacológico cuando la PA es ≥ 160/100 mmHg o en HTA grado 1 si el riesgo cardiovascular es alto. En HTA grado 1 con riesgo moderado/bajo, iniciar tratamiento tras unos meses con medidas de estilos de vida	Moderada	Fuerte a favor
El objetivo terapéutico de control en todos los pacientes es < 140/90 mmHg, y tender a alcanzar un rango de PAS de 120-129 mmHg (130-139 en enfermedad renal crónica) si < 65 años y de 130-139 mmHg si > 65 años	Moderada	Fuerte a favor

AMPA: automedida de la presión arterial; ARA-II: antagonistas de los receptores de la angiotensina ii; HTA: hipertensión arterial; IECA: inhibidores de la enzima de conversora de angiotensina; MAPA: monitorización ambulatoria de la presión arterial.

### Pruebas diagnósticas y clasificación

La HTA se define por unas cifras repetidamente elevadas de PA en la consulta clínica ≥ 140/90 mmHg^33^, ^34^. Estos valores se basan en evidencias de ensayos clínicos aleatorizados, que muestran disminución de la morbimortalidad CV con la reducción de las cifras de PA en sujetos hipertensos. Las mediciones de la PA deben obtenerse de manera estandarizada en cualquier ámbito de medición (clínico, hogar o monitorización ambulatoria)^35^, ^36^, ^37^, ^38^.

Las guías norteamericanas del American College of Cardiology/American Heart Association (ACC/AHA) definieron la HTA como cifras de PA en la consulta ≥ 130/80 mmHg, basados en el estudio SPRINT, unos pocos metaanálisis y algunos estudios observacionales^39^, ^40^. Sin embargo, esos estudios se centraron selectivamente en pacientes de alto riesgo o con enfermedad CV, por lo que la definición de HTA no es generalizable. Además, un análisis de simulación muestra que si en España se implementaran las guías norteamericanas en vez de las europeas^33^, el número de nuevos hipertensos aumentaría unos 5 millones y habría 1,4 millones más de nuevos candidatos a tratamiento farmacológico, una situación difícilmente asumible para el sistema sanitario^41^.

La PA tomada en la consulta de forma convencional es un buen método de cribado (sospecha) de HTA^33^. El ensayo HOPE 4 ha mostrado que el cribado comunitario y la detección precoz de los factores de riesgo vascular realizado por trabajadores no sanitarios colaborando con médicos de atención primaria se asocia a una mejora en el control de la HTA^42^. Sin embargo, no hay apenas evidencias que determinen los períodos o los grupos de pacientes en los que se deba realizar el cribado de la HTA. La United States Preventive Services Task Force (USPSTF)^43^ propone que el cribado de la HTA se realice en adultos de edad ≥ 40 años o con mayor riesgo de desarrollar HTA, mediante una evaluación anual, y cada 3-5 años en las personas menores de 40 años que no estén en alto riesgo de desarrollar HTA. Los factores de riesgo de HTA considerados en la USPSTF son: sobrepeso u obesidad, PA normal-alta o ser afroamericano^43^. Según la guía europea, la decisión de cribar cada 3 o 5 años depende de que las cifras iniciales se encuentren en el rango normal u óptimo, respectivamente^33^.

Las mediciones fuera de la consulta mediante MAPA o mediante automedición de la PA (AMPA) tienen un poder pronóstico CV y cerebrovascular superior al de las mediciones en la consulta^44^, ^45^, ^46^, ^47^, ^48^, ^49^ y evitan el sobrediagnóstico relacionado con el frecuente fenómeno de bata blanca y el consiguiente probable sobretratamiento^50^. Por ello, son útiles para la confirmación diagnóstica de HTA ante cifras elevadas de PA en la consulta. Además, permiten el diagnóstico de hipertensión enmascarada (hipertensión en el hogar o en la vida diaria y no en la consulta), así como evaluar mejor la hipertensión resistente^33^. La MAPA tiene, como ventaja adicional, la obtención de cifras nocturnas de la PA y del estado *dipping* (grado de caída nocturna de la PA respecto al día) y es la estrategia diagnóstica y de indicación terapéutica más coste-efectiva para la mayoría de los adultos en el ámbito de atención primaria^51^. Sin embargo, la AMPA está más disponible en AP^52^ y es un sustituto aceptable de la MAPA^33^, ^39^. Si la MAPA o la AMPA no estuvieran disponibles, las determinaciones protocolizadas de la PA en la consulta pueden utilizarse para diagnosticar la HTA^33^. Estas recomendaciones también están recogidas en otras guías clínicas, como la guía británica NICE^53^ y la de la Sociedad Internacional de Hipertensión^54^ (aunque en esta última, en consonancia con la guía europea^33^, la toma de la PA fuera de la consulta no es considerada como imprescindible para el diagnóstico, sino más bien un complemento a la toma en la consulta).

Una opción adicional a la AMPA, si no existe disponibilidad de aparatos de medida por parte de los pacientes, sería la toma de PA en consulta sin presencia de personal sanitario^38^, en cuyo caso las cifras diagnósticas serían similares a las de la AMPA. Esta forma de toma de PA también es recomendada en las guías europeas, aunque se advierte de su menor evidencia para predecir complicaciones^33^. Además, este tipo de medición no es considerada factible por muchos médicos de familia en España^52^. Finalmente, para el diagnóstico de la HTA se consideran distintos umbrales dependiendo del método de medida de la PA: *a)* 140/90 mmHg para la toma de la PA en consulta; *b)* 135/85 mmHg para los valores domiciliarios de la AMPA y los valores diurnos de la MAPA; *c)* 130/80 mmHg con medias promediadas en las 24 h del día en la MAPA, y *d)* 120/70 mmHg para los valores nocturnos de la MAPA^33^.

En función de las cifras de PAS y PAD, la PA se clasifica en: óptima (PAS < 120 mmHg y PAD < 80 mmHg), normal (PAS 120-129 y PAD 80-84 mmHg), normal-alta (PAS 130-139 o PAD 85-89 mmHg), HTA grado 1 (PAS 140-159 o PAD 90-99 mmHg), HTA grado 2 (PAS 160-179 o PAD 100-109 mmHg), HTA grado 3 (PAS >180 o PAD >110 mmHg) e hipertensión sistólica aislada (PAS >140 mmHg y PAD < 90 mmHg).

### Medidas de estilos de vida

Las medidas de estilos de vida son útiles para retrasar o complementar el tratamiento farmacológico del paciente hipertenso y aportan beneficios de salud más allá de su impacto sobre la PA. El control del peso corporal, la práctica de ejercicio aeróbico regular (p. ej., al menos 30 min de ejercicio dinámico moderado 5-7 días a la semana), la reducción del consumo de sal (menos de 2 g de sodio o 5 g de sal) o la reducción del consumo de alcohol (< 14 unidades por semana en varones y < 8 unidades por semana en mujeres) o de sal (< 5 g/día) reducen las cifras de PA^33^, ^39^, ^54^, ^55^, ^56^.

Para reducir la PA, incluyendo la PA ambulatoria, se recomienda también una dieta saludable equilibrada (dieta mediterránea o dietas similares), con consumo elevado de verduras, legumbres, fruta fresca, pescado, nueces y ácidos grasos insaturados (aceite de oliva), bajo consumo de carne roja y grasa saturada, y consumo de lácteos bajos en grasa^57^, ^58^, ^59^, ^60^. Por último, aunque el abandono del tabaco no tiene efectos objetivos sobre el descenso de la PA, se recomienda su cesación al paciente hipertenso fumador para reducir su riesgo CV^33^, ^61^.

## Tratamiento farmacológico

### Reducción del riesgo de enfermedad vascular y de mortalidad

El tratamiento farmacológico de la HTA disminuye la morbimortalidad vascular (enfermedad cerebrovascular, cardiopatía isquémica, mortalidad CV y mortalidad total), si bien la evidencia procede principalmente de ensayos clínicos que incluían predominantemente a pacientes mayores o con alto riesgo CV^33^, ^39^, ^40^.

### Niveles de presión arterial para iniciar el tratamiento farmacológico

Como norma general, se recomienda iniciar inmediatamente el tratamiento farmacológico cuando la PA es ≥ 160/100 mmHg^33^, ^62^, ^63^. En presencia de HTA de grado 1 (140-159/90-99 mmHg) se debe iniciar el tratamiento de forma inmediata si el riesgo CV es alto o coexiste enfermedad CV, enfermedad renal o daño orgánico mediado por la HTA^33^, ^64^, ^65^, y si el riesgo es moderado o bajo, sin enfermedad CV, renal, o daño orgánico, se debería iniciar el tratamiento si tras 3-6 meses de modificación de estilos de vida, la PA sigue sin estar controlada^33^, ^64^, ^65^. En los pacientes mayores de 80 años^66^, y sin fragilidad, se recomienda iniciar el tratamiento si la PA sistólica es ≥ 160 mmHg^33^.

En casos de PA normal-alta (130-139/85-89 mmHg), se puede considerar el tratamiento si el paciente tiene riesgo CV muy alto por enfermedad CV, especialmente enfermedad coronaria^33^, ^67^.

Para el cálculo del riesgo CV se recomienda utilizar la tabla SCORE para países europeos con bajo riesgo CV, como España, o tablas locales validadas^56^, ^68^.

### Objetivos terapéuticos de presión arterial

Existe práctica unanimidad en recomendar cifras de PA < 140/90 mmHg como objetivo terapéutico en todos los pacientes y siempre que el tratamiento sea bien tolerado debería dirigirse hacia cifras de 130/80 o inferiores en la mayoría de los pacientes^33^, ^63^. Específicamente, en pacientes tratados < 65 años, el objetivo es reducir la PAS a 130 mmHg y hacia valores de 120-129 mmHg si se toleran, salvo en enfermedad renal crónica (ERC), cuyo objetivo es alcanzar una PAS < 140 mmHg y tender hacia valores de 130 mmHg si se toleran. En pacientes tratados ≥ 65 años el objetivo terapéutico se establece en el rango de 130-139 mmHg en todos los pacientes (incluyendo los >80 años no frágiles), intentando los valores más bajos si se toleran^33^, ^62^, ^69^. El objetivo terapéutico de la PAD es alcanzar un rango de 70-79 mmHg en todos los pacientes. El límite de seguridad de la PAS, por debajo del cual el riesgo supera al beneficio, está en torno a los 120 mmHg^70^. Estas consideraciones afectan a todos los sujetos, incluyendo a aquellos con diabetes o con enfermedad CV previa^33^.

### Fármacos

La mayoría de los pacientes requerirán terapia con fármacos antihipertensivos además de medidas de estilos de vida para alcanzar un control óptimo de su PA. Aunque se dispone de 5 grandes clases de fármacos (IECA, ARA-II, betabloqueantes, calcioantagonistas y diuréticos)^33^, el efecto sobre la morbimortalidad es similar entre ellos^62^, ^68^, ^71^.

Para alcanzar un buen control de la PA, más aún en los grados 2 o 3 de HTA, se suelen necesitar 2 o más fármacos^33^, ^54^, ^72^. Como la regulación de la PA depende de muchos mecanismos, en la mayoría de las personas con HTA las combinaciones de fármacos que actúan por diferentes mecanismos reductores de la PA son especialmente eficaces. Además, algunos estudios han mostrado que la asociación farmacológica en un solo comprimido es más eficaz para reducir la PA que la monoterapia o la combinación de fármacos por separado^33^, ^73^, ^74^. Esas son las razones por las que la guía europea de HTA recomienda el inicio del tratamiento farmacológico mediante combinación de 2 fármacos (preferiblemente en un solo comprimido) en una gran parte de los pacientes hipertensos^33^, ^75^; la excepción son los pacientes muy mayores, los mayores con fragilidad, y los pacientes con HTA grado 1 en bajo riesgo CV^33^. Por su parte, las guías ACC/AHA recomiendan el tratamiento combinado con 2 fármacos de diferente clase en la HTA estadio 2 (≥ 140/90 mmHg en esta guía)^39^.

Respecto a las clases de fármacos utilizados en las combinaciones, las guías europeas recomiendan un IECA o ARA-II con un calcioantagonista o un diurético tiazídico o similar a las tiazidas (clortalidona, indapamida) como terapia inicial para la mayoría de los pacientes^33^. Para aquellos que requieran 3 fármacos, se recomienda una combinación de un IECA o ARA-II con un calcioantagonista y un diurético tiazídico o similar. Respecto a los diuréticos tiazídicos y análogos tiazídicos son menos efectivos como fármacos antihipertensivos para los pacientes con un filtrado glomerular (FG) disminuido (< 45 ml/min) y son ineficientes cuando el FG es < 30 ml/min. En estos casos, los diuréticos de asa, como furosemida o torasemida, deben reemplazar a las tiazidas o análogos tiazídicos para lograr el efecto antihipertensivo^33^. Por otra parte, los IECA y ARA-II reducen más la albuminuria que otros fármacos y retrasan la progresión de la ERC, diabética o no diabética, siendo los fármacos de elección en la persona con diabetes, con o sin nefropatía^33^, ^67^. En cuanto a los betabloqueantes, se recomienda que se utilicen cuando haya una indicación específica para su uso (p. ej., angina, tras un infarto de miocardio o en insuficiencia cardíaca (INC)con fracción de eyección reducida), o cuando se requiera el control de la frecuencia cardíaca. Por otra parte, persiste la contraindicación de asociar betabloqueantes con calcioantagonistas no dihidropiridínicos (diltiazem y verapamilo), así como la asociación de IECA con ARA-II o cualquiera de estos con aliskiren.

Aunque el tratamiento inicial combinado suele ser bien tolerado por los pacientes, y puede mejorar la adherencia terapéutica y el control de la PA^33^, ^76^, no se dispone de evidencia suficiente de beneficio en términos de morbimortalidad CV ni sobre los efectos secundarios; así lo comentan los autores de la más reciente revisión de las guía NICE^53^. Además, las evidencias que van generando los más recientes y potentes ensayos proceden de sujetos con alto riesgo CV (SPRINT)^77^ o con diabetes (ACCORD)^78^ y no son generalizables a sujetos de menor riesgo. Por último, el tratamiento combinado de entrada podría suponer una excesiva medicalización de los pacientes hipertensos. Por ello consideramos necesaria más evidencia antes de una pronunciación categórica para recomendar la combinación de fármacos como tratamiento inicial de la HTA.

Por último, la pandemia de la COVID-19 ha generado un debate sobre la discontinuidad de los fármacos que inhiben el sistema renina-angiotensina (iSRA) en pacientes hipertensos con esta enfermedad. Aunque la evidencia disponible es escasa, una revisión reciente y un estudio poblacional de casos y controles indican que los iSRA no incrementan el riesgo de la infección por el SARS-CoV-2 ni empeoran el curso clínico de esta enfermedad infecciosa^79^, ^80^. Como posible explicación de estos resultados, puede mencionarse que, aunque en los pacientes hipertensos existe un incremento de la enzima conversora de angiotensina ii (ECA2), que se sabe que facilita la entrada del virus en las células, es también una enzima de acción antiinflamatoria y, por otro lado, se inhibe la ECA, que tiene un marcado papel proinflamatorio. Sin embargo, se necesitan más estudios que investiguen los posibles efectos positivos de los iSRA^81^. Varias sociedades científicas y grupos de expertos, entre ellos el Grupo de Trabajo en Hipertensión Arterial de la Sociedad Española de Medicina de Familia y Comunitaria^82^ y la Sociedad Española de Hipertensión/Liga Española para la Lucha contra la Hipertensión Arterial^83^, han comunicado que no se dispone de evidencia suficiente para cambiar las directrices de tratamiento de los pacientes con HTA que presenten COVID-19 o se encuentren en riesgo de padecerla.

### Adherencia al tratamiento

Es muy importante detectar la falta de adherencia al tratamiento farmacológico antihipertensivo, pues es una de las causas más frecuentes del bajo nivel de control de la HTA^33^. Se han descrito intervenciones y uso de nuevas tecnologías, dirigidas a mejorar la adherencia terapéutica en la HTA, que han demostrado ser efectivas en la mejora de la adherencia al tratamiento y del control de la PA^84^, ^85^, ^86^, ^87^.

## Dislipidemia

### Prueba diagnóstica

La prueba recomendada para el cribado de la dislipidemia es la determinación del colesterol total, debido a su relación con la mortalidad CV, aunque debe acompañarse de la valoración del cHDL, ya que permite calcular mejor el riesgo CV. Aunque las cifras de colesterol se interpretan en función de la estratificación del riesgo CV, se suele considerar como hipercolesterolemia una cifra de colesterol total > 200 mg/dl, mientras que el efecto protector del cHDL se considera a partir de los 50 mg/dl y actuaría como factor de riesgo por debajo de los 45 mg/dl.

No hay evidencias suficientes para establecer un rango de edad y una periodicidad determinados para determinar el colesterol sérico en población sana, por lo que la actitud más razonable es incluirlo en cualquier análisis de sangre solicitado al paciente, con una periodicidad mínima de 4 años y a partir de los 18 años.

### Reducción del colesterol con los fármacos hipolipidemiantes

Las estatinas son, entre los diferentes fármacos hipolipidemiantes, los que más evidencias disponen en la reducción de la enfermedad CV y con una excelente relación riesgo/beneficio en población de riesgo^88^, ^89^. Existe una relación lineal entre los miligramos de cLDL reducidos con estatinas y la reducción de la enfermedad CV, y se ha cuantificado que descensos de 1 mmol/l (39 mg/dl) de cLDL determinan una reducción de los episodios CV mayores (infarto de miocardio e ictus mortal o no mortal, y recibir un tratamiento de revascularización) de un 21% y se sugiere que reducciones de 2-3 mmol/l reducirían el riesgo un 40-50%^90^.

Además, de los episodios CV mayores, las estatinas también reducen la mortalidad total, la mortalidad CV, la mortalidad coronaria y los ictus isquémicos^91^, pero sin resultados en la prevención de los ictus hemorrágicos^92^.

Se ha demostrado que los beneficios CV ocurren en diferentes grupos de población. Entre estos se encuentran los pacientes con o sin enfermedad CV, con ictus, personas con diabetes, en varones y mujeres, en mayores de 65 años e, incluso, en pacientes con riesgo CV bajo^91^, ^93^, ^94^. La extensa población en la que las estatinas han demostrado su eficacia hace que, prioritariamente, se recomiende el tratamiento en los grupos con mayor riesgo CV: enfermedad CV, personas con diabetes, riesgo alto mediante tablas de riesgo o pacientes con un cLDL muy elevado.

### Fármacos

En general, las estatinas constituyen la piedra angular del tratamiento de la dislipidemia y los otros fármacos hipolipidemiantes tienen su principal indicación cuando existe intolerancia a las estatinas o combinados con estas.

Los estudios que comparan el efecto de añadir otro fármaco hipolipidemiante a una población tratada con estatinas no han tenido demasiado éxito. Así, la combinación de estatinas con fibratos o niacina no incrementa el beneficio conseguido con la estatina sola. Sin embargo, en el estudio IMPROVE-IT^95^, la adición de ezetimiba a simvastatina produjo beneficios adicionales tras un síndrome coronario agudo y redujo el riesgo de la variable primaria (compuesta por muerte CV, infarto de miocardio, angina inestable, revascularización coronaria o ictus) en un 6% a los 7 años de seguimiento. Estos resultados serían equivalentes a los beneficios esperados al extrapolar a la relación lineal, descrita en el apartado de la reducción del riesgo CV, los miligramos de cLDL reducidos al asociar la ezetimiba. Un reciente análisis de subgrupos del IMPROVE-IT mostró que el mayor beneficio se observa en pacientes diabéticos y en pacientes de muy alto riesgo CV^96^.

Los estudios de intervención con los inhibidores de la PCSK9 han demostrado una reducción añadida de episodios vasculares no fatales, consistentes con sus efectos reductores en cLDL y la duración de los ensayos clínicos^97^, ^98^.

En el Further Cardiovascular Outcomes Research with PCSK9 Inhibition in Subjects with Elevated Risk (FOURIER)^97^, el tratamiento con evolocumab en combinación con estatinas de moderada/alta intensidad en pacientes con enfermedad CV establecida, consiguió una reducción del 15% en el objetivo primario compuesto por muerte, infarto de miocardio, ictus, ingreso por angina inestable o revascularización coronaria en aproximadamente 2 años de seguimiento, con independencia de la concentración basal de cLDL.

Posteriormente, en el Evaluation of Cardiovascular Outcomes After an Acute Coronary Syndrome During Treatment With Alirocumab (ODYSSEY)^98^ se consiguió en pacientes con un síndrome coronario reciente un descenso similar del objetivo compuesto por muerte coronaria, infarto de miocardio no fatal, ictus isquémico fatal/no fatal o angina inestable que requiere hospitalización en el brazo de tratamiento con alirocumab, con un mayor beneficio absoluto en los pacientes con cLDL > 100 mg/dl.

Diferentes subanálisis de los estudios FOURIER y ODYSSEY han aportado nuevas evidencias de los beneficios vasculares de los inhibidores de PCSK9 en diferentes situaciones clínicas, como el paciente polivascular, especialmente con enfermedad arterial periférica^99^, ^100^ y con concentraciones elevadas de lipoproteína(a)^101^, ^102^.

Cabe destacar que los inhibidores de PCSK9 también han demostrado efectos positivos en la composición y regresión de la placa de ateroma^103^, no incrementan la incidencia de diabetes, ni empeoran el metabolismo hidrocarbonado^104^, ni presentan efectos adversos en la función cognitiva^105^, ^106^ ni aumentan el riesgo de cataratas^107^ o de cáncer^108^. Sin embargo, su perfil de seguridad deberá confirmarse en estudios con un seguimiento a largo plazo. Actualmente, las indicaciones financiadas con cargo al Sistema Nacional de Salud de los inhibidores de la PCSK9 son en los siguientes grupos:

– Pacientes con enfermedad CV establecida (cardiopatía isquémica, enfermedad cerebrovascular isquémica y enfermedad arterial periférica) no controlados con la dosis máxima tolerada de estatinas (cLDL > 100 mg/dl).
– Pacientes con hipercolesterolemia familiar no controlados con la dosis máxima tolerada de estatinas (cLDL > 100 mg/dl).
– Cualquiera de los pacientes de los grupos anteriores que sean intolerantes a las estatinas o en los que las estatinas están contraindicadas y cuyo nivel de cLDL > 100 mg/dl.

#### Ácidos grasos omega-3

Tal como se comenta en el documento del CEIPV 2020, el efecto de los ácidos grasos omega-3 en la prevención CV es controvertido, existiendo evidencias a favor y en contra^109^, ^110^, ^111^, ^112^ y se podrían dar diferentes explicaciones a cada una de las posiciones.

En el Reduction of Cardiovascular Events with Icosapent Ethyl-Intervention Trial (REDUCE-IT)^113^ el uso de altas dosis de etilo de icosapento (4 g/día) en comparación con placebo se acompañó de una reducción significativa del RR del 25% de episodios vasculares graves en sujetos con enfermedad CV estable o diabetes y concentraciones de cLDL< 100 mg/dl y de triglicéridos entre 150 y 499 mg/dl. En un subanálisis posterior de los resultados en función de los terciles de trigliceridemia, los autores concluyen que los beneficios vasculares del etilo de icosapento están relacionados principalmente con el riesgo basal y otros efectos no dependientes de los triglicéridos^114^.

En la tabla 4 podemos ver las categorías de riesgo CV y los objetivos de control lipídico propuestos por la reciente guía para el manejo de las dislipidemias de la Sociedad Europea de Cardiología (ESC) y de la Sociedad Europea de Arteriosclerosis, y adaptada por el CEIPV 2020 (en vías de publicación). Los aspectos más importantes a resaltar son los siguientes:

– Los objetivos de cLDL serían los mismos, independientemente del territorio vascular afectado, y lo importante es conseguir una reducción del cLDL ≥ 50%.
– En referencia a los objetivos terapéuticos, todos aquellos pacientes con enfermedad CV establecida se considerarán de muy alto riesgo CV con un objetivo de cLDL < 55 mg/dl y reducción > 50%. Los pacientes con hipercolesterolemia familiar deben ser considerados de alto riesgo y el objetivo sería un cLDL < 70 mg/dl y una reducción del cLDL > 50%. Si además presentan otro factor de riesgo asociado, se considerarán de muy alto riesgo CV, con un objetivo de cLDL < 55 mg/dl y una reducción > 50%.
– Los objetivos en pacientes con riesgo CV moderado-bajo son controvertidos y la evidencia no es tan consistente como en las categorías de muy alto o alto riesgo CV. En los de riesgo CV moderado, hay que tener en cuenta los modificadores de riesgo para decidir si precisan o no tratamiento con estatinas. En los pacientes de bajo riesgo, se recomienda únicamente cambios de estilo de vida.
– Se recomienda par la mayor parte de la población general cambios de estilo de vida y lo razonable sería mantener el cLDL < 130 mg/dl.

**Tabla 4:** Categorías de riesgo cardiovascular y objetivos de control lipídico propuestos por la guía para el manejo de las dislipidemias de la Sociedad Europea de Cardiología (ESC) y de la Sociedad Europea de Arteriosclerosis y adaptada por el Comité Español Interdisciplinar de Prevención Vascular (CEIPV 2020)

Categorías de riesgo vascular	Objetivo primario cLDL	Objetivos secundarios^c^	
		ApoB	C-no-HDL
*Riesgo muy alto* Enfermedad vascular (clínica/por imagen) SCORE ≥ 10% ERC Severa (FG < 30 ml/min) DM y LOD: ≥ 3 FRV mayores o inicio temprano de DM1 de larga duración (> 20 años) HF con EV o con otro FRV mayor	< 55 mg/dl y reducción > 50% < 40 mg/dl (con recurrencia del evento vascular < 2 años)	< 65 mg/dl	< 85 mg/dl
*Riesgo alto* SCORE ≥ 5% y < 10% Un FRV marcadamente aumentado, en particular CT > 8 mmol/l (> 310 mg/dl), cLDL > 4,9 mmol/l (cLDL > 190 mg/dl) o presión arterial ≥ 180/110 mmHg ERC moderada (FG 30-59 ml/min) DM sin LOD, con una duración de DM ≥ 10 años con algún factor asociado HF sin otros FRV mayores	< 70 mg/dl y reducción > 50%	< 80 mg/dl	< 100 mg/dl
*Riesgo moderado*^a^ SCORE ≥ 1% y < 5% Pacientes jóvenes (DM1 < 35 años; DM2 < 50 años) con DM < 10 años sin otros FRV	< 130 mg/dl		< 160 mg/dl
*Riesgo bajo*^b^ SCORE < 1%	< 130 mg/dl^b^	-	-

LOD: lesión de órgano diana: definido como oligoalbuminuria, retinopatía o neuropatía.

ApoB: apolipoproteína B; C-no-HDL: colesterol no HDL; cLDL: colesterol unido a lipoproteínas de baja densidad; CT: colesterol total; DM: diabetes mellitus; DM1: DM tipo 1; DM2: DM tipo 2; ERC: enfermedad renal crónica; EV: enfermedad vascular; FG: filtrado glomerular; FRV: factores de riesgo vascular; HF: hipercolesterolemia familiar; LOD: lesión de órgano diana; PA: presión arterial; SCORE: Systematic Coronary Risk Estimation; TG: triglicéridos.

a En los pacientes de riesgo vascular moderado se han de tener en cuenta los modificadores de riesgo para decidir si precisan o no tratamiento con estatinas.

b Se recomienda para la mayor parte de la población general cambios de estilo de vida y lo razonable serían mantener el cLDL < 130 mg/dl.

c La ApoB se recomienda como una alternativa al cLDL, particularmente en personas con niveles altos de TG, DM, obesidad, síndrome metabólico o niveles muy bajos de cLDL. Se puede usar, si está disponible, como la medida principal para la detección, el diagnóstico y el tratamiento, y puede preferirse al C-no-HDL en estos pacientes, si bien su baja disponibilidad en nuestro medio hace que el C-no-HDL sea la opción más operativa.

En la tabla 5 se recoge, de forma resumida, la calidad de la evidencia y la fuerza de la recomendación sobre el manejo de la dislipidemia.

**Tabla 5:** Recomendaciones en dislipidemia

	Calidad de la evidencia	Fuerza de la recomendación
*Prueba diagnóstica*		
Las pruebas recomendadas para el cribado son colesterol total y cHDL	Moderada	Fuerte a favor
Periodicidad del cribado cada 4 años	Baja	Fuerte a favor
Recomendada a partir de los 18 años en ambos sexos	Baja	Fuerte a favor
*Tratamiento farmacológico*		
Las estatinas son los fármacos de elección para el tratamiento de la dislipidemia	Alta	Fuerte a favor
En caso de no alcanzar los objetivos terapéuticos con estatinas a dosis plenas o de intolerancia a las estatinas, se recomienda la ezetimiba	Moderada	Fuerte a favor
En caso de pacientes con enfermedad cardiovascular establecida, o pacientes con HFHo o con HFHe, todos ellos con la dosis máxima tolerada de estatinas y un cLDL > 100 mg/dl, o bien cualquiera de esos pacientes con intolerancia a las estatinas y con cLDL > 100 mg/dl, se recomienda la utilización de inhibidores del PCSK9 (evolocumab o alirocumab)	Moderada	Fuerte a favor
Los objetivos y los criterios para introducir los fármacos hipolipidemiantes dependen del cLDL y de la estratificación del riesgo cardiovascular	Moderada	Fuerte a favor

cHDL: colesterol unido a lipoproteínas de alta densidad; cLDL: colesterol unido a lipoproteínas de baja densidad; HFHe: hipercolesterolemia familiar heterocigota; HFHo: hipercolesterolemia familiar homocigota.

## Diabetes mellitus tipo 2

### Importancia epidemiológica y clínica del problema

La prevalencia de diabetes mellitus (DM) en España en población mayor de 18 años es de un 6,9%, mayoritariamente conocida (79,5%), tratada con fármacos (85,5%) y controlada entre los pacientes tratados (69%)^115^. Si bien en otro estudio^116^ el porcentaje de control fue del 56,1%, sin individualizar objetivos de HbA1c y el 60,5% individualizando. Otro estudio español, el estudio Di@bet.es, realizado con sobrecarga oral de glucosa (SOG), también con base poblacional y en mayores de 18 años, duplica la prevalencia de la diabetes (13,8%; IC del 95%, 12,8-14,7%), de los que, la mitad (6%; IC del 95%, 5,4-6,7%) no estaba diagnosticada previamente^117^. Un metaanálisis de 55 estudios europeos^118^ ha demostrado un 5,46% (IC del 95%, 4,7-6,1) de diabetes no diagnosticada. La DM tipo 2 (DM2) es el tipo más común de diabetes y representa el 90% de los casos de diabetes en todo el mundo^119^. Con relación a la incidencia de DM2, en población española mayor de 18 años es de 11,6 casos/1.000 personas-año (IC del 95%, 11,1–12,1), con una incidencia de DM no conocida de 7.9 casos/1.000 personas-año (IC del 95%, 5,3-8,1)^120^. Los factores de riesgo asociados a una mayor incidencia de DM2 fueron: ser varón (OR 2,7, IC del 95%, 1,6-4,5), tolerancia alterada a la glucosa (TAG) (OR 7,9, IC del 95%, 4,0-15,5), glucemia basal alterada (GBA) (OR 11,7, IC del 95%, 5,9-23,3), TAG + GBA (OR 48,8, IC del 95%, 17,1-139,8), obesidad (IMC > 30 kg/m^2^) (OR 2,3, IC del 95%, 1,1-4,6), obesidad central (≥ 94 cm en hombres y ≥ 80 en mujeres) (OR 3,4, IC del 95%, 1,5-7,8) e historia familiar de DM (OR 2,3, IC del 95%, 1,6-3,3)^120^.

Un estudio poblacional en Cataluña^121^ basado en revisión de 286.791 historias clínicas electrónicas muestra el siguiente perfil de la persona con DM2: edad media de 68,2 años; 6,5 años de evolución de la enfermedad; HbA1c del 7,15%; PA de 137,2/76,4 mmHg; cLDL de 112,5 mg/dl e IMC de 29,6 kg/m^2^; un 15,4% fumadores (hombres 24%, mujeres 6%); un 11,3% con antecedentes de enfermedad isquémica de miocardio; un 6,5% de enfermedad cerebrovascular, y un 2,9% con enfermedad arterial periférica. Respecto al grado de control, de los pacientes en prevención primaria CV, solo un 12,9% tenía buen control integral (HbA1c <  = 7%, PA <  = 130/80 mmHg y cLDL < 130 mg/dl) y un 12,1% en prevención secundaria (HA1c <  = 7%, PA <  = 130/80 mmHg y cLDL < 100 mg/dl). El 22% estaba tratado solo con medidas no farmacológicas y un 23,4% recibía insulina.

La diabetes supone un incremento del riesgo de mortalidad CV de aproximadamente 2 veces con respecto a la persona sin diabetes. Además, también está asociada con una importante mortalidad prematura causada por algunos cánceres, enfermedades infecciosas, causas externas, suicidios y trastornos degenerativos, independientemente de los principales factores de riesgo^122^.

La prediabetes, definida como GBA (glucemia en ayunas 110-125 mg/dl), y la TAG (glucemia 140-200 mg/dl tras SOG con 75 g) se asocian con modestos incrementos en el riesgo de enfermedad CV. Así, la GBA tuvo un RR de 1,20 (IC del 95%, 1,12-1,28) para valores entre 110-125 mg/dl y de 1,18 (IC del 95%, 1,09-2,10) entre 100 y 125 mg/dl, y la TAG, un RR de 1,20 (IC del 95%, 1,07-1,34)^123^.

### Prueba diagnóstica

#### Prueba recomendada para el cribado y valores para el diagnóstico

Existen varias estrategias para el cribado de la DM2 y de la prediabetes, como el cribado oportunista, mediante la realización de glucemia en poblaciones con mayor riesgo, los cuestionarios o las escalas de riesgo, que permiten identificar subgrupos de población en los que estaría recomendado realizar el cribado. La prediabetes y la diabetes se diagnostican mediante la medición de la glucemia basal en ayunas, la medición a las 2 h tras SOG o mediante la medición de la HbA1c (tabla 6).

**Tabla 6:** Criterios diagnósticos de prediabetes y diabetes

	Glucemia basal (mg/dl)	HbA1c (%)	Glucemia al azar + síntomas	SOG-2 h (mg/dl)
Prediabetes	100-125^a^	5,7-6,4	NC	140-199^b^
Diabetes mellitus	≥ 126	≥ 6,5	≥ 200	≥ 200

HbA1c: hemoglobina glucosilada; NC: no considerado; SOG-2 h: sobrecarga oral de glucosa o medición de glucemia a las 2 h tras 75 g de glucosa.

a Glucemia basal alterada (GBA).

b Tolerancia alterada a la glucosa (TAG).

La HbA1c, comparada con la glucemia basal, proporciona pequeñas mejoras en la predicción del riesgo CV en pacientes no diagnosticados de diabetes^124^. Sin embargo, en las personas con diabetes, la HbA1c presenta una asociación más fuerte con el riesgo de microangiopatía (retinopatía y nefropatía) y enfermedades CV que la media de las glucemias^125^. Por lo tanto, es una prueba tan útil como la glucemia basal para el diagnóstico y mejor que la glucemia para valorar el grado de control de la DM2.

Una revisión sistemática concluyó que el cribado no reduce la mortalidad en un seguimiento a 10 años y subraya la necesidad de más estudios que determinen la efectividad del tratamiento de la diabetes detectada por cribado^126^. El estudio ADDITION observó que el cribado frente al no cribado no fue superior en la reducción del riesgo de mortalidad total *(hazard ratio* [HR]: 1,06; IC del 95%, 0,90-1,25), mortalidad CV (HR: 1,02; IC del 95%, 0,75-1,38) o mortalidad relacionada con la diabetes (HR: 1,26; IC del 95%, 0,75-2,10)^127^.

Se han publicado evidencias de que el tratamiento (estilo de vida o farmacológico) sobre los estados prediabéticos diagnosticados por cribado se asocia a reducción del riesgo de progresión a diabetes^128^, ^129^, ^130^, ^131^, ^132^, ^133^. Pero la evidencia disponible no permite confirmar ni descartar posibles efectos perjudiciales del cribado. Una revisión sistemática no observó reducción en la mortalidad total y CV en personas tratadas con fármacos tras ser diagnosticadas de prediabetes por cribado^134^. En una revisión Cochranne^135^ no se demuestra que la dieta sola o el ejercicio físico por sí solo modifiquen la incidencia de diabetes o sus complicaciones en pacientes con riesgo de desarrollar diabetes. Pero la adición de dieta y ejercicio sí previene o retrasa la incidencia de DM2 en pacientes con TAG.

Otra revisión sistemática^136^, que incluyó 138 estudios, concluye que la HbA1c no tiene suficiente sensibilidad ni especificidad como prueba de cribado y que la glucemia en ayunas es específica pero poco sensible. Las diferentes pruebas empleadas en el cribado identifican poblaciones muy diferentes y sus indicadores de validez no son buenos, por lo que muchas personas estarán innecesariamente tratadas o falsamente despreocupadas, dependiendo de la prueba empleada (tabla 7). La prevalencia de prediabetes fue del 27% con criterios Organización Mundial de la Salud y del 54% con criterios de la American Diabetes Association (ADA), con gran variabilidad según la prueba empleada (glucemia basal, SOG o HbA1c). Este metaanálisis pone en duda la validez de las políticas de «cribar y tratar» en diabetes. Con estas evidencias, no puede recomendarse el cribado poblacional de DM2, salvo en pacientes con factores de riesgo asociados y con antecedentes familiares de diabetes, recomendando realizar la prueba de cribado aprovechando otra analítica.

**Tabla 7:** Validez de las pruebas de cribado en diabetes mellitus tipo 2

	Glucemia basal (mg/dl)^a^		HbA1c (%)^b^	
	Todos los estudios (n = 23)	Estudios con bajo riesgo de sesgo (n = 18)^c^	Todos los estudios (n = 21)	Estudios con bajo riesgo de sesgo (n = 18)^c^
Sensibilidad	0,25 (0,19-0,32)	0,24 (0,17-0,32)	0,49 (0,40-0,58)	0,47 (0,37-0,58)
Especificidad	0,94 (0,92-0,96)	0,95 (0,93-0,97)	0,79 (0,73-0,84)	0,81 (0,74-0,86)
Falsos negativos	0,75 (0,68-0,81)	0,76 (0,86-0,83)	0,51 (0,42-0,60)	0,53 (0,42-0,63)
Falsos positivos	0,06 (0,04-0,08)	0,05 (0,03-0,07)	0,21 (0,16-0,27)	0,19 (0,14-0,26)
Área bajo la curva	0,72	0,73	0,71	0,71

Entre paréntesis se presentan los intervalos de confianza.

HbA1c: hemoglobina glucosilada.

a Glucemia basal como prueba de cribado y glucemia basal alterada como prueba de referencia.

b HBA1c como prueba de cribado y test de sobrecarga oral de glucosa como prueba de referencia.

c Se excluyen los estudios con alto riesgo de sesgo y se incluyen solo los de bajo riesgo de sesgo. Resultados tomados, traducidos y adaptados de Bertomeu-González et al.^22^.

### Periodicidad recomendada

La periodicidad de la determinación de la glucemia basal en la persona sin diabetes no tiene evidencias para establecer una recomendación. Sin embargo, la glucemia suele añadirse habitualmente en el contexto de la detección o el seguimiento de otros factores de riesgo CV. El test FINDRISC permite identificar a sujetos de alto riesgo de presentar DM2 si la puntuación es mayor de 15 puntos, evitando la glucemia como prueba de cribado (estudio DE-PLAN)^137^ y ha sido validado en español^138^.

### Medidas no farmacológicas

Una intervención dirigida a promover, tras el cribado, el abordaje multifactorial temprano intensivo en personas con DM2 se asoció con una pequeña, pero no significativa, reducción en la incidencia de episodios CV y muerte^139^. Las intervenciones en estilo de vida basadas en alcanzar y mantener un 7% de pérdida de peso y en realizar 150 o más minutos por semana de actividad física moderada-intensa^140^ y, en menor medida, la metformina producen pérdida de peso y previenen o retrasan la incidencia de diabetes^141^. También la educación diabetológica individual en pacientes con DM2 reduce la HbA1C^142^, pero sin evidencias de reducción de la enfermedad CV. Por otro lado, las intervenciones pueden perder efectividad con el tiempo. En un metaanálisis^143^, la intervención dietética y de ejercicio produjo reducciones de HbA1c a los 3 y 6 meses, pero no a los 12 y 24 meses. Existe una calidad moderada-alta de la evidencia en la recomendación de modificación de estilos de vida (dieta o ejercicio) a las personas con diabetes.

### Fármacos

Existen algunas incertidumbres sobre la eficacia y la seguridad CV de los fármacos antidiabéticos, así como sobre la idoneidad de un tratamiento intensivo. Tras los buenos resultados con el UKPDS y el STENO-2, y los decepcionantes resultados de los estudios RECORD, PROactive, ACCORD, ADVANCE y VADT, desde el año 2008 la Food and Drug Administration (FDA) y la European Medicines Agency (EMA) exigen estudios de seguridad CV para los nuevos fármacos hipoglucemiantes. Sin embargo, los nuevos estudios de seguridad CV han puesto de manifiesto beneficios de prevención con los nuevos fármacos antidiabéticos.

#### Seguridad cardiovascular

Si bien no ha habido un gran estudio diseñado para evaluar los efectos de la metformina sobre la morbimortalidad CV, al ser el fármaco más empleado en el tratamiento de la DM2 en práctica clínica y en muchos estudios, sí se han publicado numerosos metaanálisis mostrando su beneficio en prevención CV^144^, ^145^, ^146^, ^147^, ^148^, ^149^. En pacientes en prevención secundaria CV, la metformina, en un metaanálisis sobre 40 estudios incluyendo 1.066.408 pacientes^31^, redujo un 33% la mortalidad por cualquier causa, un 19% la mortalidad CV y un 17% la incidencia de nuevos eventos CV. Asimismo, la asociación de metformina con inhibidores de la dipeptidil peptidasa 4 (iDPP4) con relación a la de metformina y sulfonilureas redujo la incidencia de eventos CV y mortalidad (HR 0,71 [0,56-0,90]) para evento CV no mortal, la mortalidad CV (HR 0,58 [0,41-0,82]) y la mortalidad por todas las causas (HR 0,72 [0,59-0,87])^146^, confirmado con otros estudios^147^, ^149^, si bien otro estudio comparando la combinación metformina con sulfonilureas de 2.ª-3.ª generación respecto a otras combinaciones no encontró un aumento de riesgo CV^150^. En monoterapia, el beneficio preventivo CV de la metformina respecto a las sulfonilureas^151^ es claro, por lo que la metformina es el tratamiento inicial de elección en los pacientes con DM2.

No existen evidencias del beneficio CV de las sulfonilureas. La mayoría de los estudios muestran un aumento del riesgo de morbimortalidad CV^152^, ^153^, ^154^ muchas veces mediado por la mayor incidencia de hipoglucemias^155^ o en otros casos un efecto CV neutro^156^. Si bien puede haber diferencias entre las sulfonilureas de 1.ª, 2.ª y 3.ª generación^150^, donde glimepirida y gliclazida se asociaron con menor riesgo CV que la glibenclamida^157^.

Los estudios en monoterapia frente a placebo, con iDPP4, TECOS (sitagliptina), EXAMINE (alogliptina) y SAVOR-TIMI (saxagliptina) muestran una no inferioridad en seguridad CV, si bien en el SAVOR-TIMI se observó un aumento de la hospitalización por INC. En el estudio CARMELINA^158^, la hospitalización por INC y los episodios renales graves fueron similares con linagliptina o placebo. En el estudio CAROLINA^159^ el riesgo de eventos CV fue similar comparando linagliptina con glimepirida.

Con relación a los inhibidores del cotransportador de sodio-glucosa tipo 2 (iSGLT2), el estudio EMPA-REG (empagliflozina) en pacientes en prevención secundaria CV mostró una reducción significativa de la morbimortalidad CV (HR 0,86; IC del 95%, 0,74-0,99) al igual que el estudio CANVAS (canagliflozina) (HR 0,86; IC del 95%, 0,75-0,97), si bien este con un 35% de pacientes en prevención primaria CV. El estudio CREDENCE^160^ es el primero que utiliza un objetivo primario renal, con una reducción del mismo a favor de canagliflozina del 30% (HR: 0,70; IC del 95%, 0,59-0,82, p = 0,00001). Los resultados de los objetivos secundarios CV también fueron favorables para el tratamiento con canagliflozina. El estudio se interrumpió prematuramente por beneficio significativo en el grupo de tratamiento activo. En el estudio DECLARE^161^, dapagliflozina no alcanzó una reducción significativa del objetivo CV principal (8,8% vs. 9,4%; HR: 0,93; IC del 95%, 0,84-1,03; p = 0,17), aunque si alcanzó el criterio de no inferioridad frente a placebo. Tampoco hubo diferencias en la mortalidad CV (HR 0,98; IC del 95%, 0,82-1,17) ni en la mortalidad por cualquier causa (6,2% vs. 6,6% [HR 0,93; IC del 95%, 0,82-1,04]). Sí hubo una reducción de ingresos por INC (HR 0,73; IC del 95%, 0,61-0,88) y de eventos de causa renal (4,3% vs. 5,6%; HR 0,76 [IC del 95%, 0,67-0,87]). En otro análisis del estudio DECLARE TIMI 58 con dapagliflozina^162^, sí se observó una reducción de la morbimortalidad CV, en pacientes en prevención secundaria CV (HR 0,84; IC del 95%, 0,72-0,99; p = 0,039) pero no en prevención primaria CV (HR 1,00; IC del 95%, 0,88-1,13; p = 0,97).

El estudio DAPA-HF^163^ es el primero de un iSGLT2 (dapagliflozina) realizado en población con y sin DM2, con INC con fracción de eyección < 40% (NYHA II-IV). Se observó una reducción del objetivo principal (muerte CV, hospitalización por INC o necesidad tratamiento por vía intravenosa para INC) (6,3% vs. 21,2% (HR 0,74; IC del 95%, 0,60-0,85; p< 0,001), así como de la primera hospitalización por INC (10,0% vs. 13,7%; HR 0,70; IC del 95%, 0,59-0,83), como de muerte CV (9,6% vs. 11,5% [HR 0,82; IC del 95%, 0,69-0,98]) y de muerte por cualquier causa (11,5% vs. 13,9% [HR 0,83; IC del 95%, 0,71 a 0,97]). Un metaanálisis^164^ observa que en conjunto los iSGLT2 reducen la mortalidad por cualquier causa (HR 0,70; IC del 95%, 0,59-0,83), la mortalidad CV (HR 0,43; IC del 95%, 0,36-0,53) y la tasa de infartos de miocardio (HR 0,77; IC del 95%, 0,63-0,94), pero no la de ictus (HR 1,09; IC del 95%, 0,86-1,38).

Con relación a los agonistas del receptor del péptido similar al glucagón tipo 1 (arGLP1)^165^, ^166^, ^167^, ^168^, ^169^, ^170^, ^171^, los estudios LEADER (liraglutida), SUSTAIN (semaglutida), REWIND (dulaglutida) y HARMONY (albliglutida) mostraron reducción significativa del *mayor adverse cardiovascular events* (MACE, en inglés) de incidencia de morbimortalidad CV entre el 13% (liraglutida) y el 26% (semaglutida). Por el contrario, los estudios LIXA (lixisenatida) y EXSCEL (exenatida-LAR) se mostraron neutros, no aumentando ni reduciendo la incidencia de eventos CV. El estudio PIONEER 6 (57) con semaglutida por vía oral observó una reducción del 51% de la muerte CV (HR 0,49; 0,27-0,92; p = 0,03) y del 49% de la mortalidad de cualquier causa (HR 0,51; 0,31-0,84; p = 0,008). Pero sin diferencias significativas (no inferioridad) en el objetivo principal o MACE.

Un metaanálisis^172^ muestra que las terapias basadas en incretinas (iDPP4 y arGLP1) no incrementan el riesgo de eventos CV y que la liraglutida sí los reduce significativamente. Otro metaanálisis^173^ evidencia con los arGLP1, reducciones significativas en mortalidad por cualquier causa (HR: 0,88; IC del 95%, 0,79-0,97), mortalidad CV (HR: 0,84; IC del 95%, 0,74-0,96) e infarto de miocardio (HR: 0,90; IC del 95%, 0,80-1,00). No se observa beneficio en ictus (HR: 0,89; IC del 95%, 0,76-1,04) ni en INC (HR: 0,92; IC del 95%, 0,81-1,06). Otro metaanálisis^174^ revisando 8 estudios con iSGLT2 o arGLP1 incluyendo a 77.242 personas con diabetes, evidencia una reducción de la morbimortalidad CV de 11% para iSGLT2 (HR: 0,89, IC del 95%, 0,83-0,96; p = 0,001) y de 12% para los arGLP1 (HR: 0,88, IC del 95%, 0,84-0,94; p < 0,001), con mayor eficacia en previsión secundaria CV (HR: 0,86, IC del 95%, 0,80-0,93; p = 0,002) y sin significación estadística en prevención primaria (HR: 0,93; IC del 95%, 0,83-1,04; p = 0,20). Se observó también una reducción en la progresión de la enfermedad renal tanto con iSGLT2 (HR: 0,62; IC del 95%, 0,58-0,67; p < 0,001) como con arGLP1 (HR: 0,82; IC del 95%, 0,75-0,89; p < 0,001) pero solo los iSGLT2 redujeron el riesgo de empeoramiento del FG, enfermedad renal terminal o muerte de causa renal (HR: 0,55; IC del 95%, 0,48-0,64; p < 0,001). Los iSGLT2 redujeron la hospitalización por INC un 31% (HR; 0,69; IC del 95%, 0,61-0,79; p < 0,001), pero no así los arGLP1-RA (HR 0,93; IC del 95%, 0,83-1,04; p = 0,20).

Otro metaanálisis^175^ revisando 27 estudios que incluyeron a 56.004 pacientes, incluyendo asimismo los estudios ELIXA, LEADER, SUSTAIN-6, EXSCEL, HARMONY, REWIND, y PIONEER 6, concluye que los arGLP1 tiene beneficios demostrados de prevención CV, así como de prevención de indicadores de enfermedad renal en pacientes con DM2 (HR: 0,88 [IC del 95%, 0,81-0,96; p = 0,003) para muerte CV, 0,84 (0,76-0,93; p < 0·0001) para ictus fatal o no fatal, 0,91 (0,84-1,00; p = 0,043) para infarto de miocardio fatal o no fatal; HR: 0,88 (0,83-0,95; p = 0,001) para la mortalidad por todas las causas, y HR: 0,91 (0,83-0,99; p = 0,028), para ingresos hospitalarios por INC. Y una reducción del 17% (0,83, 0,78-0,89; p< 0,0001) del objetivo compuesto de prevención de daño renal, fundamentalmente debido a la reducción de la excreción urinaria de albúmina. No hubo incremento del riesgo de hipoglucemias graves, pancreatitis o cáncer de páncreas.

### Intervención intensiva sobre la glucemia

En relación con los posibles beneficios CV tras un control glucémico intensivo, distintos metaanálisis observaron que el control intensivo de la glucemia (HbA1c < 7%) redujo el riesgo de eventos CV en un 9% (HR: 0,91; IC del 95%, 0,84-0,99), fundamentalmente gracias a la reducción del 15% en el riesgo de infarto de miocardio, y no se observó disminución ni de la mortalidad total ni de la CV^176^, ^177^. Otros metaanálisis^178^, ^179^ subrayan que el control glucémico intensivo no solo no se asocia a reducción de mortalidad, sino que conlleva un incremento de hipoglucemias graves (RR: 2,39; IC del 95%, 1,71-3,34). Un metaanálisis más reciente^180^ concluye que, frente al tratamiento estándar, el tratamiento intensivo reduce el riesgo de infarto de miocardio no fatal (RR: 0,90; IC del 95%, 0,83-0,96), el de mortalidad CV (RR: 1,00; IC del 95%, 0,90-1,11) o el de mortalidad por cualquier causa (RR: 0,72; IC del 95%, 0,46-1,14), pero no el de ictus no fatal (RR: 0,96; IC del 95%, 0,86-1,07). Un control intensivo de la diabetes puede estar justificado en pacientes con retinopatía o microalbuminuria. Un reciente estudio poblacional realizado en España muestra que el control intensivo en personas ancianas (> 75 años) incrementa el riesgo de hipoglucemias^181^. Sin embargo, otra cohorte española de 5.016 pacientes con DM2 mayores de 70 años observó que los pacientes con HbA1c< 7% presentaron menor incidencia de morbimortalidad CV que los que tenían HbA1c 7-8% o mayor del 8%^182^.

Un concepto importante en la prevención de eventos CV en personas con DM2 es el de legado glucémico, entendido como el efecto del control glucémico precoz en los primeros años del diagnóstico de DM2 en la prevención del desarrollo de complicaciones a largo plazo. En un estudio de cohortes retrospectivas^183^, con inclusión de 34.737 pacientes, los pacientes que tenían un HbA1c inferior al 6,5% en el primer año tras el diagnóstico de DM2 presentaron, a los 13 de años de seguimiento, un menor riesgo de eventos microvasculares y macrovasculares, y los pacientes con HbA1c ≥ 7% presentaron un mayor riesgo de mortalidad, un aumento del 29% en pacientes con una HbA1c entre el 7 y el 8% y del 32% si la HbA1c era ≥ 9.

### Intervenciones multifactoriales

Respecto a la eficacia de una intervención multifactorial, los estudios UKPDS^184^, ^185^ y STENO-2^186^ mostraron la importancia del control de todos los factores de riesgo CV. En un metaanálisis, las intervenciones multifactoriales frente al control estándar mostraron una reducción del ictus no fatal (RR: 0,53; IC del 95%, 0,32-0,87), pero no de infarto de miocardio no fatal (RR: 0,66; IC del 95%, 0,38-1,03), de mortalidad CV (RR: 0,72; IC del 95%, 0,46-1,14) o de mortalidad por cualquier causa (RR: 0,82; IC del 95%, 0,64-1,05).

### Estrategias terapéuticas

Según las guías de práctica clínica más relevantes, en general se recomienda un tratamiento escalonado que empieza por metformina y va incorporando más fármacos orales (sulfonilureas, glitazonas, iDPP4 o iSGLT2) o inyectables (insulinas o arGLP1) si persiste el mal control. La guía del Primary Care Diabetes Europe (PCDE)^187^ recomienda inicio de tratamiento combinado para evitar la inercia y mejorar el grado de control y la guía de semFYC^188^ recomienda no esperar más de 3 meses, tras el inicio o modificación terapéutica, para realizar una analítica e intensificar el tratamiento si no se alcanza el control y la adherencia es adecuada.

Asimismo, se proponen algoritmos adaptados a perfiles de personas con DM2 si coexiste ERC, obesidad o enfermedad CV. En líneas generales, se debe observar un objetivo de control menos estricto en pacientes ancianos o con comorbilidades asociadas o con pobre calidad de vida, entendiendo por un control estándar una HbA1c del 7-8%. En pacientes con ERC se deben evitar fármacos contraindicados; en pacientes con obesidad se deben contemplar fármacos que ayuden a perder peso y en pacientes con enfermedad CV se deben priorizar fármacos que reduzcan la morbimortalidad CV.

Pero a pesar de los nuevos avances terapéuticos el grado de control no presenta grandes avances. Así, en un estudio poblacional sobre registros en historia clínica electrónica en práctica clínica en Cataluña^189^, en el período 2007-2013, analizando, respectivamente, a 257.072 y 343.969 personas con diabetes, la proporción de pacientes con HbA1c menor del 7% fue del 54,9% en 2007 y del 55,2% en 2013. Otro estudio en Reino Unido^190^, realizado en el período 2012-2016, en 164 centros de atención primaria, mostraba un 46,7% de pacientes con HbA1c menor del 7%.

Entre las causas^191^, destacan la falta de adherencia terapéutica por parte del paciente y la inercia clínica^190^ o falta de intensificación del tratamiento por parte del profesional sanitario. Diversos criterios se emplean para valorar la adherencia, como son la persistencia en la prescripción de la medicación o el porcentaje entre las dosis de fármaco dispensadas y las dosis necesarias en un período *(medication possesion ratio* o MPR en inglés)^192^. Una revisión sistemática^193^ mostró una MPR del 75,3% (IC del 95%, 68,8%-81,7%), con una proporción de pacientes con buena adherencia del 67,9% (IC del 95%, 59,6%-76,3%). Un estudio en Alemania analizando 1.201 consultas de atención primaria observó un mayor porcentaje de discontinuidad del tratamiento en los pacientes tratados con sulfonilureas que en los tratados con iDPP4 (49% vs. 39%; HR 0,74, IC del 95%, 0,71-0,76) con una incidencia de eventos CV un 26% menor en los pacientes tratados con iDPP4^194^. Otro metaanálisis muestra que en personas con DM2 una buena adherencia se asocia con una reducción del riesgo de hospitalización, así como de la mortalidad por todas las causas^195^. También debe destacarse que la adherencia a las guías de práctica clínica en atención primaria es baja^196^.

La falta de intensificación del tratamiento, a pesar de no alcanzar los objetivos establecidos, es otra causa importante de falta de control glucémico. Un metaanálisis revisando 53 artículos^197^ concluyó que el tiempo medio para la intensificación terapéutica fue de un año (rango 0,3-7,2), hubo mayor inercia cuando el número de fármacos era mayor y menor cuando los niveles de HbA1c eran más altos. Un estudio español^197^ observó que no se realizaba intensificación en uno de cada 5 pacientes con mal control y que el 26% de los pacientes con HbA1c mayor del 7% permanecían sin intensificar su tratamiento después de 4 años de seguimiento.

En la tabla 8 se recoge, de forma resumida, la calidad de la evidencia y la fuerza de la recomendación sobre el manejo de la DM.

**Tabla 8:** Recomendaciones en diabetes mellitus tipo 2

	Calidad de la evidencia	Fuerza de la recomendación
*Prueba diagnóstica y control glucémico*		
Realizar cribado poblacional de DM2	Alta	Fuerte en contra
La prueba de cribado recomendada es la glucemia basal	Moderada	Débil a favor
La HbA1c es preferible para monitorizar el control de la glucemia en la persona con DM2	Moderada	Fuerte a favor
Un control adecuado de la diabetes debería estar entre un 7 y un 8% de HbA1c	Alta	Fuerte a favor
Deben calcularse objetivos individualizados, más estrictos en pacientes jóvenes sin complicaciones (6,5%) y menos estrictos en pacientes mayores con comorbilidades importantes (8%). Se recomienda el uso de la app para *smartphones* (A1c Calculator Application) para el cálculo de objetivos individualizados^a^	Moderada	Débil a favor
*Tratamiento farmacológico*		
La metformina es el fármaco recomendado para el tratamiento inicial de la diabetes, salvo contraindicación o intolerancia	Alta	Fuerte a favor
La glibenclamida no debe emplearse por el mayor riesgo de hipoglucemias y de eventos CV respecto a otras opciones	Alta	Fuerte a favor
En prevención secundaria (pacientes con evento cardiovascular), el fármaco de elección asociado a metformina será un ISGLT2 (empaglifozina, canaglifozina) o un arGLP1 (liraglutida, semaglutida, dulaglutida) asociado o no a cualquier otro antidiabético, salvo contraindicación o intolerancia	Alta	Fuerte a favor
Debe evitarse la asociación de iDPP4 y arGLp1, por compartir el mismo mecanismo de acción	Moderada	Débil a favor
La mejor estrategia de tratamiento es una intervención multifactorial de la diabetes	Alta	Fuerte a favor
*Recomendaciones con menor evidencia*		
La periodicidad mínima de la determinación de la glucemia en población de riesgo es cada 4 años	Baja	Débil a favor
El test FINDRISC permite evitar la glucemia en el cribado de la prediabetes y diabetes partir de un punto de corte (≥ 15 puntos)	Baja	Débil a favor
*No existe suficiente evidencia para su recomendación*		
Control intensivo a todos los pacientes con diabetes	Alta	Fuerte en contra

a Actualmente, ya se dispone de una *app* para *smartphones* (A1c Calculator Application), que es gratuita. Disponible en: https://play.google.com/store/apps/details?id=com.crocodil.software.a1ccalc&hl=es

## Tratamiento antiagregante

### Importancia epidemiológica y clínica del problema

Se ha documentado que la utilización de ácido acetilsalicílico (AAS) en prevención secundaria alcanza un grado de utilización razonablemente alto, alrededor del 94%^198^, en consonancia con la abundante evidencia existente. Sin embargo, en prevención primaria existen incertidumbres sobre su balance beneficio-riesgo y las recomendaciones de las guías de práctica clínica se han modificado según han ido apareciendo nuevas evidencias, lo que se traduce en mucha heterogeneidad en el uso de AAS en pacientes que no han presentado una enfermedad CV, incluidos los pacientes de alto riesgo CV y los pacientes diabéticos. En el estudio EUROASPIRE III en prevención primaria, el tratamiento antiagregante se había prescrito en el 22% de los pacientes de alto riesgo sin enfermedad CV y en el 28,2% de los diabéticos^199^.

Los antiagregantes por vía oral actualmente disponibles en España son los siguientes: los inhibidores de la síntesis de tromboxano: AAS (el más utilizado) y el trifusal; inhibidores de la fosfodiesteresa: dipiridamol, y los inhibidores de la activación plaquetaria mediada por ADP (inhibidores del receptor P2Y12) ticlopidina, clopidogrel, prasugrel, ticagrelor.

### Revisión de la evidencia

#### Prevención secundaria

El tratamiento antiagregante con AAS a una dosis de entre 75 y 150 mg/día en pacientes con enfermedad CV previa (prevención secundaria) produce una reducción significativa de todos los episodios vasculares mayores y de la mortalidad CV y total^200^. En pacientes con alergia o intolerancia al AAS, el clopidogrel es la alternativa de elección^201^.

En el caso específico de la prevención de la recurrencia del ictus, se puede recomendar como primera línea de tratamiento tanto AAS 50-300 mg como clopidogrel 75 mg, trifusal 300 mg o la combinación AAS (25 mg)/dipiridamol (200 mg en liberación retardada), 2 veces al día (en España solo está comercializado dipiridamol de liberación rápida)^202^.

#### Prevención primaria

Se han publicado diferentes metaanálisis en prevención primaria de la enfermedad CV. Baigent et al.^203^ mostraron una reducción relativa de un 12% de todos los eventos CV (AAS 0,51% frente a control 0,57% por año; p = 0,0001), que se debió, en su mayoría, a una reducción relativa de un 23% para el infarto de miocardio no fatal (AAS 0,18% frente a control 0,23% por año; p < 0,0001). De Berardis et al.^204^ analizaron los resultados según el sexo y observaron que el AAS reduce significativamente el riesgo de infarto de miocardio en varones en un 43%, sin encontrar diferencias en mujeres, mientras que el AAS reducía el riesgo de ictus en mujeres en un 25% de una manera significativa, sin encontrar diferencias en varones.

No hay evidencia de que el AAS reduzca significativamente la mortalidad por enfermedad CV ni la mortalidad total, aunque, en un metaanálisis, la reducción observada alcanzó el límite de la significación (RR: 0,94; IC del 95%, 0,88-1,00; p = 0,05)^205^. Un metaanálisis más reciente confirma la reducción no significativa de la mortalidad CV (RR 0,94, 0,83-1,05), con una reducción significativa de IAM (0,85, 0,73-0,99) y de ictus isquémico (0,81, 0,76-0,87), pero con un incremento importante del riesgo de hemorragias graves (1,43, 1,30-1,56)^206^.

### Hemorragias

En el metaanálisis de Baigent et al. se observó un aumento significativo de riesgo de hemorragia mayor extracraneal con AAS (RR: 1,54; IC del 95%, 1,30-1,82; p < 0,0001), en su mayoría no fatal^203^. Respecto a los otros metaanálisis, uno de ellos mostró aumento significativo del riesgo de ictus hemorrágico (RR: 1,36; IC del 95%, 1,01-1,82; p = 0,04), hemorragia mayor (RR: 1,66; IC del 95%, 1,41-1,95; p < 0,00001) y hemorragia gastrointestinal (RR: 1,37; IC del 95%, 1,15-1,62; p = 0,0003)^205^, mientras que otro mostró el mismo incremento de riesgo, tanto para el ictus hemorrágico como para la hemorragia mayor^207^. El metaanálisis de Seshasai et al.^208^ mostró un exceso de riesgo del 70% del total de las hemorragias y del 30% de las hemorragias no menores.

### Ácido acetilsalicílico y diabetes

*Prevención de eventos isquémicos.* Ninguno de los 5 metaanálisis que se centraban en pacientes diabéticos^204^, ^209^, ^210^, ^211^, ^212^ mostró un efecto protector significativo del AAS en la prevención de eventos CV. Todos los resultados favorecían al AAS (sin alcanzar significación estadística) y, en el metaanálisis de Butalia et al., la reducción de eventos resultó en el límite de la significación (p = 0,05)^209^.

*Hemorragias*. Stavrakis et al.^210^ mostraron un aumento del riesgo de hemorragias mayores con AAS cuando utilizaban un estimador agrupado de efectos fijos (RR: 2,51; IC del 95%, 1,11-5,70; p = 0,028); sin embargo, no se encontraron diferencias entre ambos grupos cuando el estimador era de efectos aleatorios (RR: 3,02; IC del 95%, 0,48-18,86; p = 0,24)^210^. Del resto de metaanálisis, aun observando una tendencia a mayores hemorragias en el grupo con AAS, ninguno de ellos resultó estadísticamente significativo^204^, ^209^, ^211^, ^212^.

### Ácido acetilsalicílico e insuficiencia renal

Un análisis de subgrupos *post hoc* del estudio Hypertension Optimal Treatment (HOT)^213^ mostró que el beneficio del AAS era mayor en pacientes con hipertensión y ERC, con un aumento progresivo del beneficio en la reducción de los eventos CV y la mortalidad total en los pacientes con un FG ø 45 ml/min/1,73 m^2^ ^214^.

Un metaanálisis^215^ sobre el uso de antiagregantes en pacientes con ERC analizó el efecto del AAS en el contexto de un síndrome coronario agudo y también en pacientes con enfermedad coronaria estable o pacientes de alto riesgo. Concretamente, en este último grupo se observó una reducción significativa del infarto de miocardio fatal o no fatal, no así en el ictus o en la mortalidad CV o por todas las causas, con un aumento de las hemorragias menores. Los resultados fueron inciertos por la baja calidad de los estudios incluidos.

### Pacientes tratados mediante revascularización coronaria

Respecto a la doble antiagregación tras una revascularización coronaria, la estrategia a seguir dependerá del contexto clínico, de la urgencia y del tipo de intervención (intervención coronaria percutánea [ICP] o cirugía de *bypass*), así como del tipo de *stent* implantado en su caso^216^, ^217^.

*Pacientes coronarios estables tratados mediante una intervención coronaria percutánea electiva.* Se recomiendan 6 meses de doble antiagregación con AAS y clopidogrel, independientemente del tipo de *stent* metálico implantado. El AAS se recomienda como tratamiento de por vida.

Pacientes que han presentado un síndrome coronario agudo sin elevación del ST (SCASEST) tratados mediante una intervención coronaria percutánea. Se recomienda doble antiagregación durante un año con AAS 75-100 mg más un inhibidor del receptor plaquetario P2Y12, siempre y cuando no exista una contraindicación formal (p. ej., exceso de riesgo de hemorragia). Se recomienda ticagrelor 90 mg, 2 veces al día, en pacientes con un moderado o alto riesgo isquémico (como haber tenido elevación de troponinas), independientemente del tratamiento inicial. Se recomienda prasugrel 10 mg, una dosis diaria, en los pacientes con anatomía coronaria conocida tratados con ICP. Se recomienda clopidogrel 75 mg diarios en los pacientes que no pueden recibir ticagrelor o prasugrel, o que requieran adicionalmente anticoagulación oral (triple terapia).

Pacientes que han presentado un infarto agudo de miocardio con elevación del ST (IAMCEST) tratados mediante una intervención coronaria percutánea^218^, ^219^. Doble antiagregación durante un año con AAS 75-100 mg más un inhibidor del receptor plaquetario P2Y12, siempre y cuando no exista una contraindicación formal (p. ej., exceso de riego de hemorragia). Se puede utilizar cualquiera de estas opciones terapéuticas: clopidogrel, ticagrelor o prasugrel. Un metaanálisis que comparó los resultados de los pacientes que recibieron doble antiagregación durante < 12 meses, 12 meses exactos o más de 12 meses^220^ mostró que los pacientes que discontinuaban antes de los 12 meses después de una ICP con *stent* liberador de fármacos tenían significativamente menos riesgo de hemorragia sin un mayor riesgo de eventos isquémicos que los que alcanzaban 12 o más meses. Por contra, los pacientes que seguían un tratamiento con doble antiagregación más de 12 meses observaron una reducción de las complicaciones isquémicas, especialmente la trombosis del *stent* y la incidencia de nuevos infartos; sin embargo, aumentaban las complicaciones hemorrágicas con riesgo de muerte.

*Pacientes con revascularización miocárdica mediante cirugía* (bypass *coronario).* Se recomienda el AAS a dosis baja de forma indefinida, o el clopidogrel en caso de intolerancia al AAS, en los casos de cardiopatía isquémica estable. Por el contrario, en los pacientes con SCA, la doble antiagregación ha probado su eficacia en reducir el riesgo isquémico con independencia de la forma de revascularización. La doble antiagregación en los pacientes con enfermedad CV crónica y estable, ya sea coronaria o de otra localización, no es más eficaz que el AAS solo en la reducción de nuevos episodios CV^221^.

*Utilización de los inhibidores de la bomba de protones.* Se recomienda la utilización de inhibidores de la bomba de protones a todos los pacientes con doble antiagregación.

### Pacientes con diagnóstico de fibrilación auricular y enfermedad coronaria estable

*Pacientes con un CHA2DS2-VASc* *> 2 y un riesgo bajo de hemorragia (HAS-BLED <* *2).* Implantación de *stent* no farmacoactivo: doble antiagregación y un anticoagulante por vía oral (triple terapia), durante un mes, seguido de doble terapia con un anticoagulante oral y un antiagregante (AAS o clopidogrel) durante un año. Después un anticoagulante por vía oral de por vida.

*Implantación de* stent *farmacoactivo.* Doble antiagregación y un anticoagulante por vía oral (triple terapia) de 3 a 6 meses, dependiendo del tipo de *stent,* seguido de doble terapia con un anticoagulante por vía oral y un antiagregante (AAS o clopidogrel) durante un año. Después un anticoagulante por vía oral de por vida.

*Pacientes con un CHA2DS2-VASc* *> 2 y un riesgo alto de hemorragia (HAS-BLED* *> 3).* Se recomienda colocar un *stent* no farmacoactivo y triple terapia de 2-4 semanas, y después solo anticoagulante de por vida.

### Pacientes con diagnóstico de fibrilación auricular y síndrome coronario agudo

En los pacientes con un HAS-BLED ≤ 2 se debe considerar la triple terapia durante 6 meses, seguida de un anticoagulante por vía oral más AAS o clopidogrel hasta 12 meses.

En los pacientes con un HAS-BLED ≥ 3 se debe considerar la triple terapia durante un mes, seguida de AAS o clopidogrel y un anticoagulante oral.

En la tabla 9 se recoge, de forma resumida, la calidad de la evidencia y la fuerza de la recomendación sobre antiagregación.

**Tabla 9:** Recomendaciones en antiagregación

	Calidad de la evidencia	Fuerza de la recomendación
AAS en prevención secundaria. El tratamiento con AAS a dosis bajas se debe utilizar en todos los pacientes diagnosticados de enfermedad coronaria, ictus o accidente isquémico transitorio, o enfermedad arterial periférica sintomática, de forma indefinida	Alta	Fuerte a favor
AAS en prevención primaria*.* No se recomienda el uso de AAS de forma sistemática en prevención primaria, incluidas las personas con diabetes. De forma individualizada, y valorando la preferencia del paciente, se podría valorar su utilización	Baja	Débil a favor
Clopidogrel como alternativa al AAS*.* El tratamiento con clopidogrel está indicado en casos de alergia o intolerancia al AAS	Moderada	Fuerte a favor
Doble antiagregación en pacientes coronarios estables tratados mediante ICP electiva. Se recomienda AAS y clopidogrel durante un mes si se trata de un *stent* convencional *(bare metal stent)* y durante 6 meses si se trata de un *stent* liberador de fármaco *(drug-eluting stent).* Después de este período se recomienda AAS de forma indefinida	Alta	Fuerte a favor
Doble antiagregación en pacientes que han presentado un SCASEST tratados mediante una ICP con *stent* liberador de fármaco*.* Se recomienda AAS 75-100 mg y un inhibidor del receptor plaquetario P2Y12 durante 12 meses: – Ticagrelor 90 mg 2 veces al día, en pacientes de moderado-alto riesgo isquémico – Prasugrel 10 mg una dosis diaria, en pacientes que van a proceder a la realización de revascularización percutánea – Clopidogrel 75 mg una dosis diaria, en pacientes que no pueden recibir ticagrelor o prasugrel, o que requieran anticoagulación oral adicional (triple terapia)	Alta	Fuerte a favor
Doble antiagregación en pacientes que han presentado un IAMCEST tratados mediante una IPC con *stent* liberador de fármaco. Se recomienda AAS 75-100 mg y un inhibidor del receptor plaquetario P2Y12 durante 12 meses. En este caso, se puede utilizar cualquiera de las opciones terapéuticas (clopidogrel, ticagrelor o prasugrel). En caso de necesitar anticoagulación por vía oral adicional, se debe utilizar clopidogrel	Alta	Fuerte a favor
Inhibidores de la bomba de protones. Se recomiendan en los pacientes tratados con doble antiagregación y con un riesgo alto de padecer una hemorragia gastrointestinal	Moderada	Fuerte a favor
Triple terapia (anticoagulante y doble antiagregación). Se recomienda en los pacientes con firme indicación de anticoagulación por vía oral (como la HA) durante un tiempo limitado, que dependerá del riesgo de hemorragia	Moderada	Fuerte a favor

AAS: ácido acetilsalicílico; FA: fibrilación auricular; IAMCEST: infarto agudo de miocardio con elevación del ST; ICP: intervención coronaria percutánea; SCASEST: síndrome coronario agudo sin elevación del ST.

## Cribado de la fibrilación auricular

### Importancia epidemiológica y clínica del problema

La fibrilación auricular (FA) afecta al 1-2% de la población general, más cerca del 2% debido al infradiagnóstico y a la cada vez más reconocida fibrilación subclínica^222^, ^223^. Se ha estimado, a través de un amplio estudio poblacional de la Sociedad Española de Cardiología^224^, que un 4,4% de la población española mayor de 40 años presenta FA, lo que supone que más de 1 millón de personas en España presenta FA en sus distintas formas clínicas. En muchas ocasiones, la arritmia es silente o episódica, y tarda tiempo en diagnosticarse, de modo que en muchos casos se reconoce de forma casual en pacientes asintomáticos o con escasos e inespecíficos síntomas^225^.

La complicación más grave de la FA es el ictus cardioembólico; sin embargo, los ictus relacionados con la fibrilación son, en gran medida, potencialmente prevenibles con el tratamiento anticoagulante.

### Prueba diagnóstica

El diagnóstico de la FA es electrocardiográfico.

Ya que, con raras excepciones, la fibrilación produce un pulso arrítmico, se ha planteado que la toma de pulso periférico podría ser una buena aproximación a su reconocimiento precoz. Para el cribado de la FA se han planteado distintas estrategias, de tipo oportunista o de tipo sistemático en población de riesgo (p. ej., mayores de 65 años, el grupo de mayor riesgo de presentar fibrilación y sus complicaciones) a través de la toma de pulso en la consulta, toma de presión por AMPA con dispositivos validados^226^ (avalado por el NICE)^227^ o de la realización de un electrocardiograma (ECG) directamente.

La toma de pulso (y realización de un ECG si este es irregular) es más económica, accesible y no supone ningún riesgo para el paciente ni exige dispositivos adicionales.

La detección de la FA permitiría instaurar tratamiento anticoagulante en los pacientes con riesgo embólico asociado según las recomendaciones de las guías^228^, ^229^, ^230^ y, de este modo, reducir las complicaciones tromboembólicas.

### Evidencia científica

Distintos estudios han evaluado el cribado de la FA en población de riesgo, generalmente desde atención primaria que involucra a profesionales de enfermería, médicos de familia o ambos^231^, ^232^. El estudio de mayor tamaño y con un diseño apropiado (ensayo clínico multicéntrico por conglomerados, aleatorizado y controlado) fue realizado en Reino Unido en población atendida en 50 centros de atención primaria^233^. Se distribuyó a aleatoriamente 14.802 pacientes con edad > 65 años a uno de los siguientes 3 brazos: *a)* grupo control (práctica clínica habitual); *b)* grupo de intervención mediante cribado oportunista (toma de pulso en consulta aprovechando otros motivos y ECG si el pulso era irregular), y *c)* cribado sistemático (se citaba mediante carta a los pacientes para realizar un ECG).

La variable principal del estudio fue el número de nuevos casos diagnosticados de fibrilación y se observaron diferencias significativas entre los grupos de intervención y control, de forma que, cuando se realizaba cualquier cribado, se detectaban más personas con FA que con la práctica clínica habitual (un 64% más). En cambio, no se apreciaron diferencias en la detección de FA nueva entre el cribado sistemático y el oportunista.

La estrategia oportunista mediante toma de pulso permitió detectar similar número de pacientes con FA que el cribado sistemático con ECG y se sugirió este cribado oportunista como método para incorporar a la práctica clínica en las consultas de atención primaria. Adicionalmente, esta estrategia ha probado ser coste-efectiva: el cribado oportunista anual se asocia a una menor incidencia de ictus isquémicos y a una mayor proporción de casos de fibrilación diagnosticados. El análisis de sensibilidad muestra una probabilidad del 60% de que la detección en mayores de 65 años (frecuencia anual en el modelo) sea rentable, tanto en varones como en mujeres^234^.

El estudio DOFA, ensayo clínico multicéntrico realizado en atención primaria en España^235^, sugiere que, en condiciones de práctica real, una búsqueda activa, a través de la palpación del pulso, de FA en pacientes de edad > 65 años con síntomas o signos indicativos es una estrategia más eficaz que el cribado oportunista en pacientes asintomáticos.

En atención primaria se atienden y se siguen (tanto en consulta médica como de enfermería) pacientes con uno o múltiples factores de riesgo CV, también ligados a una mayor incidencia de FA^225^. La práctica de la toma del pulso con el objetivo de detectar irregularidad puede ser una práctica con bajo coste y alta rentabilidad clínica^236^. Debe tenerse en cuenta que el uso cada vez mayor de los sistemas oscilométricos para la medición de la PA puede suponer una pérdida de oportunidad en la detección si no se toma el pulso adicionalmente.

No existe una evidencia directa de que la toma de pulso tenga un impacto en la reducción de episodios tromboembólicos, pero hay claros indicios de que esto debe ser así por diversas razones: *a)* la inequívoca relación entre FA e ictus; *b)* la fibrilación puede permanecer asintomática largo tiempo antes de diagnosticarse; *c)* en la gran mayoría de los casos, su diagnóstico condiciona la anticoagulación tras evaluar el balance de riesgo embólico frente al riesgo hemorrágico; *d)* el tratamiento ha demostrado ser muy eficaz para la prevención de ictus y otras embolias sistémicas, y *e)* la presencia de casos asintomáticos o subclínicos, por ejemplo, en pacientes portadores de ciertos dispositivos capaces de registrar la arritmia ha demostrado asociarse con un aumento de la incidencia de ictus isquémico, similar a la fibrilación clínica^223^.

Un estudio realizado en Holanda, en el que se evaluó si el cribado de FA mediante ECG portátil de una única derivación en personas mayores de 65 años coincidiendo con el período de vacunación de la gripe era coste-efectivo, concluyó que su implantación en atención primaria podría reducir costes y aumentar los años de vida ajustados por calidad^236^.

### Fármacos

El tratamiento anticoagulante por vía oral puede realizarse con agentes antivitamina K (acenocumarol, warfarina), que tienen indicación en cualquier tipo de FA (valvular o no valvular), o bien con los nuevos anticoagulantes de acción directa (dabigatrán, apixabán, rivaroxabán, edoxabán), que no precisan monitorización y han demostrado una similar eficacia a los agentes antivitamina K con menos complicaciones hemorrágicas graves, como la hemorragia intracraneal^237^. Estos agentes no están indicados en la FA valvular^228^, ^229^, ^230^.

La indicación del tratamiento anticoagulante se realiza sobre la base del riesgo embólico del paciente (estimado mediante escalas apropiadas como CHA2DS2-VASc Score cuando > 2), cuando se estima que este es superior al riesgo de hemorragia inherente a la propia anticoagulación.

### Otras recomendaciones

La European Society of Cardiology^228^ y la AHA/American Stroke Association^230^ recomiendan el cribado oportunista mediante la toma de pulso en población de riesgo (mayores de 65 años, mujeres mayores de 75 años), con un grado de recomendación i-B.

Una revisión Cochrane publicada en 2016^238^ sigue reconociendo la eficacia del cribado para el diagnóstico precoz de la FA, aunque sugiere la necesidad de futuras investigaciones para evaluar el impacto sobre los resultados clínicos, así como nuevos posibles métodos de cribado.

En octubre del 2017 se publicó un documento de consenso de la European Heart Rhythm Association (EHRA), en el que participaron la Heart Rhythm Society (HRS), la Asia Pacific Heart Rhythm Society (APHRS) y la Sociedad Latino-americana de Estimulación Cardíaca y Electrofisiología (SOLAECE)^239^. En dicho documento se establece la recomendación de cribado oportunista con toma de pulso a mayores de 65 años y plantea que se podría considerar el cribado sistemático con ECG a mayores de 75 años o personas con factores de riesgo de accidente cerebrovascular agudo.

## Conclusiones

Aunque la evidencia científica es limitada y no está documentada una relación directa entre la toma de pulso en población de riesgo y una reducción de los episodios tromboembólicos o mortalidad, parece plausible que una mayor detección de la FA conllevara un beneficio clínico significativo a medio y largo plazo. La toma de pulso está incluida en cualquier examen clínico básico, tanto en la atención primaria como en el ámbito hospitalario. Por tanto, no parece probable atribuir costes adicionales a lo que se debería hacer de forma rutinaria en situaciones muy comunes en la práctica clínica ordinaria.

A pesar de estar basadas en un único y amplio estudio (y una revisión Cochrane que lo incluye), las guías de práctica clínica actualmente vigentes recomiendan el cribado de la FA por su bajo coste y potencial beneficio clínico debido a la magnitud del problema (alta prevalencia y esta va en aumento) y de sus consecuencias (ictus como primera causa de muerte en la mujer, segunda en el varón, alta discapacidad asociada en los supervivientes, costes hospitalarios y sociosanitarios elevados) y sus bien documentadas posibilidades de prevención mediante la anticoagulación por vía oral.

En la tabla 10 se recoge, de forma resumida, la calidad de la evidencia y la fuerza de la recomendación sobre el cribado de FA.

**Tabla 10:** Recomendaciones sobre el cribado de la fibrilación auricular

Intervención	Calidad de la evidencia	Fuerza de la recomendación
Cribado oportunista de la FA mediante la toma de pulso en atención primaria en personas con 65 años o más y realización de ECG si el pulso es irregular	Moderada	Débil a favor

ECG: electrocardiograma; FA: fibrilación auricular

## Conflicto de intereses

Los autores declaran no tener ningún conflicto de intereses.
